# Electro-Hydrodynamics of Emulsion Droplets: Physical Insights to Applications

**DOI:** 10.3390/mi11100942

**Published:** 2020-10-18

**Authors:** Muhammad Salman Abbasi, Ryungeun Song, Seongsu Cho, Jinkee Lee

**Affiliations:** 1 School of Mechanical Engineering, Sungkyunkwan University, Suwon 16419, Korea; m.salman@uet.edu.pk (M.S.A.); fbsrms135@skku.edu (R.S.); jss1872@g.skku.edu (S.C.); 2 Faculty of Mechanical Engineering, University of Engineering and Technology, Lahore 54890, Pakistan; 3 Institute of Quantum Biophysics, Sungkyunkwan University, Suwon 16419, Korea

**Keywords:** electrohydrodynamics (EHD), droplets, emulsions, deformation, instabilities, breakups

## Abstract

The field of droplet electrohydrodynamics (EHD) emerged with a seminal work of G.I. Taylor in 1966, who presented the so-called leaky dielectric model (LDM) to predict the droplet shapes undergoing distortions under an electric field. Since then, the droplet EHD has evolved in many ways over the next 55 years with numerous intriguing phenomena reported, such as tip and equatorial streaming, Quincke rotation, double droplet breakup modes, particle assemblies at the emulsion interface, and many more. These phenomena have a potential of vast applications in different areas of science and technology. This paper presents a review of prominent droplet EHD studies pertaining to the essential physical insight of various EHD phenomena. Here, we discuss the dynamics of a single-phase emulsion droplet under weak and strong electric fields. Moreover, the effect of the presence of particles and surfactants at the emulsion interface is covered in detail. Furthermore, the EHD of multi-phase double emulsion droplet is included. We focus on features such as deformation, instabilities, and breakups under varying electrical and physical properties. At the end of the review, we also discuss the potential applications of droplet EHD and various challenges with their future perspectives.

## 1. Introduction

In recent years, electrohydrodynamics (EHD) of emulsion droplets has evolved in many ways as the research gained pace in this field. The field emerged with the seminal works of Taylor in 1966, who presented his leaky dielectric model (LDM) and predicted the single-phase droplet steady deformation [1,2]. Subsequently, the validation of the model was reported, and more accurate models emerged [3,4,5,6,7,8,9,10,11,12]. Recent EHD studies have explored more complex EHD interface topologies related to multiple phase emulsion droplets, and also covered the broader aspects, such as the emulsion instabilities, breakups, and particles manipulation at the emulsion interface forming novel colloidal assemblies [13,14,15,16,17,18,19,20,21,22,23,24,25,26,27,28,29,30,31,32,33,34]. Though it has been almost six decades since the research in this area commenced, it is only until recently that a few studies pertaining to the droplet EHD with applicative prospects have been reported [35,36,37,38,39,40,41,42,43,44,45,46,47,48,49,50,51,52].

**Figure 1:**
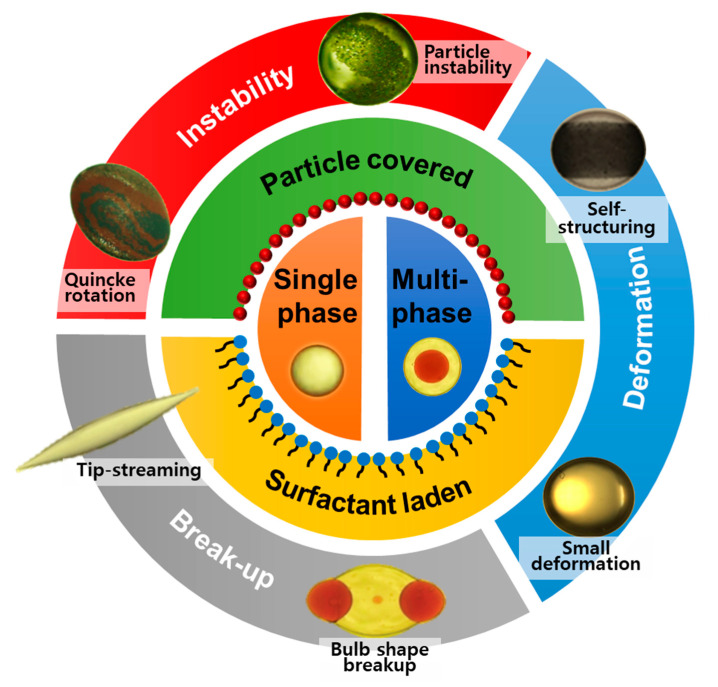
Hydrodynamics of emulsion droplets under an electric field.

This review has emphasis on the recent advancements for the essential physical insights of the droplet EHD problems. Since droplet EHD is a very broad area of research and it is not possible to cover all the aspects in a single review, here we mainly focus on the behavior of a single-phase droplet with particle-free or particle-covered interface and multi-phase (compound or double) emulsion droplets within the perspective of their deformation, instabilities, and breakups under weak and strong external stimuli of electric fields, considering only the most prominent works (See Figure 1). Here, we do not discuss the EHD of droplet coalescence, as it merits a separate review. In the latter part of the article, we also discuss the applications of droplet EHD with their challenges and future directions.

## 2. Approaches Used to Study the Droplet Electrohydrodynamics (EHD)

The approaches to study the fundamentals of droplet EHD are twofold: (i) theory and/or numerical method, and (ii) experiments. The basic analytical model is the LDM [1,2,53]. This model assumes no net charge on the interface and electroneutrality of the bulk liquids within the Stokes regime, however, the derivation of the LDM from the electro kinetic equations considering the net charge (diffuse charge or electric double layers) is still an ongoing struggle [10,54,55,56]. For numerical simulation, the governing equations of fluid flow and electro-quasi statics are implemented, and the two-phase problem is solved using different methods, such as volume of fluid (VOF) [57,58], boundary element method (BEM) [26,59,60,61], finite volume technique [62,63], level set methods (LS) [14,64,65,66], and phase field methods (PF) [67,68]. Each method has its advantages and disadvantages. Recently, CLSVOF (coupled level set and volume of fluid) method has emerged as a new approach for the interface capturing [69,70]. VOF method has its drawbacks to calculate its spatial derivatives across the interface. Then, a coupled LS and VOF approach is employed to overcome this deficiency. With its spatial gradient calculated accurately, the level-set function is smooth and continuous. For the experiments, a container (cuvette or glass-walled rectangular box) with electrodes embedded on the two opposite sides is filled with a continuous liquid and a dispersed liquid is dispensed using a micro-pipette. The high voltage is supplied across the electrodes and dynamics are captured by using cameras. In these studies, some authors have also introduced surfactants at the liquid-liquid interface [27,71,72,73,74,75,76,77,78,79,80,81,82,83,84,85,86,87,88,89]. A summary of the most prominent works related to droplet EHD are summarized in Table 1.

## 3. Background and Theory

It was a general perception before the seminal work by Taylor that the liquid droplets submerged in another immiscible liquid behave as a perfect dielectric and that their exposure to an electric field will always result in prolate deformation (elongation along the direction of the electric field) of the interface [96]. This concept was presented as the electro-hydrostatic (EHS) theory. This theory always predicted that the electric stresses act normal to the interface. However, later it was found by the experiments of Allan and Mason that all the dielectrics do not always exhibit elongation along the electric field direction and contrary to EHS theory, some show oblate deformation (elongation perpendicular to the direction of the applied electric field) [97]. Motivated by these experimental findings, Taylor in his pioneer work introduced the LDM, assuming that liquids with finite conductivities permit slight current flow through them and result in accumulation of free charge at the liquid-liquid interface [1,2]. The interaction of this free charge with the electric field results in the creation of net tangential electric stresses in addition to the normal stresses. Since hydrodynamic stresses should also exist to balance the tangential electric stresses, Taylor concluded the existence of flow circulations, which were later confirmed experimentally. Taylor’s theory predicts the small deformation of emulsion droplets under Stokes flow reasonably well. Later, Torza et al. [7] studied the response of droplets both experimentally and theoretically under DC and AC electric fields and found that Taylor’s theory underestimates the deformation for most of the cases, though the sense of the deformation observed in experiments agreed well with Taylor’s predictions. Ajayi [3] improved Taylor’s theory and calculated the droplet deformation up to a second-order approximation. His theory agreed with the experimental results up to a larger limit of droplet deformation compared to Taylor’s theory. In addition to the steady-state deformation, Esmaeeli and Sharifi [5] predicted the transient deformation of droplet.

### 3.1. Non-Dimensional Parameters

For a droplet of radius, *a*, under an unperturbed external electric field strength $E_{o}$, the characteristic velocity $U_{\infty }$ is defined as:

(1) $$U_{\infty }=\varepsilon_{2}E_{o}^{2}a/\mu_{2}$$

The droplet EHD problem depends on the non-dimensional parameters based on the liquid properties, such as conductivity ratio ($R=\sigma_{1}/\sigma_{2}$), permittivity ratio ($S=\varepsilon_{1}/\varepsilon_{2}$), and viscosity ratio ($M=\mu_{1}/\mu_{2}$) between the inside and the outside liquids. Here, the subscripts ‘1′ and ‘2′ denote the droplet and outside liquid, respectively. Non-dimensionalization of the governing equations results in other various non-dimensional parameters, such as flow Reynolds number (Re), electric Reynolds number ($Re_{E}$), and electric capillary number $\left(Ca_{E}\right)$. The Re is the ratio of inertial to viscous forces and is expressed mathematically as:

(2) $$Re=\rho_{2}aU_{\infty }/\mu_{2}$$

where $\rho_{2}$ is the density of outside liquid. LDM is valid only for low flow Reynolds number Re≤O(1) and for small droplet size.

The ratio of the viscous to the inertial force and surface tension is called the Ohnesorge Number (*Oh*), and controls the transient modes of deformation, such as monotonic and oscillating,

(3) $$Oh=\mu /\sqrt{\rho \gamma a}$$

Electric capillary number is defined as the ratio between the magnitude of electrical stresses ($\varepsilon_{2}E_{0}^{2}$) and capillary stresses (γ/a), where γ is the interfacial tension between two liquids, and controls/delineates the droplet deformation regimes, such as stable and unstable,

(4) $$Ca_{E}=\mu_{2}U_{\infty }/\gamma =\varepsilon_{2}E_{o}^{2}a/\gamma .$$

Electric Reynolds number is defined as the ratio of the charge relaxation time scale ($t_{c}=\varepsilon_{2}/\sigma_{2}$) to the time scale of charge convection by flow ($t_{h}=\mu_{2}/\varepsilon_{2}E_{0}^{2}$). A small value implies that the charge convection effects are negligible. Based on the medium properties, it is given as:

(5) $$Re_{E}=t_{c}/t_{h}=\varepsilon_{2}^{2}E_{o}^{2}/\mu_{2}\sigma_{2}$$

Another important non-dimensional number is the Saville number (*Sa*), which is the ratio of the electric capillary number and electric Reynolds number and decides the droplet breakup mode transitions,

(6) $$Sa=Re_{E}/Ca_{E}$$

LDM assumes the interface is instantly charged, i.e., the surface charge convection effects represented by electric Reynolds number ($Re_{E}$) are negligible.

### 3.2. Govening Equations of Leaky Dielectric Model (LDM)

Here, we summarize the core governing equation of LDM. Note that the equations reproduced here are for a single-phase emulsion droplet.

#### 3.2.1. Electric Field Equations and Their Solutions

For a general dynamic system, electric field ($\vec{E}$) and magnetic field ($\vec{B}$) are coupled in the governing Maxwell equations [98]. The Faraday’s law is given as:

(7) $$\frac{\partial \vec{B}}{\partial t}+\nabla \times \vec{E}=0.$$

Saville [53] showed that with small electric currents and in the absence of external magnetic field, Maxwell equations can be simplified significantly, and the Faraday’s law becomes:

(8) $$\nabla \times \vec{E}=0.$$

Equation (8) shows that the electric field is irrotational and thus, $\vec{E}=-\nabla V$, where *V* is the electric potential and the droplet is assumed to be placed in an unperturbed external electric field strength of magnitude $E_{o}$.

Gauss’s law is given as:

(9) $$Q=\nabla \cdot \left(\varepsilon \vec{E}\right)$$

where Q is the volumetric charge density and ε is the liquid permittivity.

Conservation of charge is another important governing equation given as:

(10) $$\frac{\partial Q}{\partial t}+\nabla \cdot I_{\rho }=0,$$

where $I_{\rho }$ is the current density defined as:

(11) $$I_{\rho }=\sigma \vec{E}+Q\vec{u}$$

The above equation shows that the charge movement is owing to the Ohmic conduction and the convection, respectively. Here, $\vec{u}$ is the liquid velocity and for incompressible liquids with uniform properties in each of the phases; Equations (9)–(11) are combined to give

(12) $$\frac{DQ}{Dt}+\frac{\sigma }{\varepsilon }Q=0,$$

where $D/Dt=\partial /\partial t+\vec{u}\cdot \nabla$ is the material derivative. The solution of the above equation yields:

(13) $$Q=Q_{o}\exp\left(-t/t_{c}\right).$$

The above equation shows that the volumetric charge decays by time exponentially and becomes zero in the bulk. Where, $t_{c}=\varepsilon /\sigma$ is the charge relaxation time scale or the rate of charge decay. Provided that if $t_{c}$ is much smaller than the time scale of any other process of interest, then the charge immediately accumulates at the interface and Equation (11) becomes:

(14) $$I_{\rho }=\sigma \vec{E},\;\nabla \cdot \left(\sigma \vec{E}\right)=0.$$

and for liquids with constant properties:

(15) $$\nabla \cdot \vec{E}=0.$$

As the electric field is irrotational and $\vec{E}=-\nabla V$, this equation results in:

(16) $$\nabla^{2}V=0,$$

where $\nabla^{2}$ is the Laplacian operator? For a single droplet with azimuthal symmetry, the electric potential V is only a function of r and θ in the spherical coordinates. For a droplet of radius a under an unperturbed external electric field strength $E_{o}$, the solution of Equation (16) with boundary conditions [1,7],

(17) $$V_{1}\left(0,\theta \right)\;\mathrm{should}\;\mathrm{be}\;\mathrm{bounded},\;V_{1}\left(a,\theta \right)=V_{2}\left(a,\theta \right),\;\sigma_{1}\partial V_{1}/\partial r=\sigma_{2}\partial V_{2}/\partial r,\;V_{2}\left(\infty ,\theta \right)=E_{o}r\cos\theta ,$$

leads to the electric potential at the droplet inside $V_{1}$ and droplet outside $V_{2}$ given as:

(18) $$V_{1}=\frac{3}{R+2}\left(\frac{r}{a}\right)\cos\theta \;aE_{o},$$

(19) $$V_{2}=\left[\left(\frac{r}{a}\right)-\frac{R-1}{R+2}{\left(\frac{a}{r}\right)}^{2}\right]\cos\theta \;aE_{o}.$$

The electric field at the inside and the outside of the droplet can be determined by taking the gradient of the electric potential given in Equations (18) and (19), respectively.

The Maxwell stress tensor is given as [99]:

(20) $$T_{M}=\varepsilon \vec{E}\vec{E}-\frac{1}{2}\varepsilon \vec{E}\cdot \vec{E}I.$$

The divergence of Maxwell stress tensor determines the electric force per unit volume of the liquid ($f_{E}$) that is substituted in Navier–Stokes equation. Conveniently, electric stresses can be split into normal and tangential components in a t−n coordinate system and their jumps across the interface have a direct impact on the flow field and deformation of the droplet at the steady state, respectively. The jumps in the tangential ${\left\|{\vec{T}}_{M,t}\right\|}_{12}$ and normal components ${\left\|{\vec{T}}_{M,n}\right\|}_{12}$ of electric stresses can be found by the evaluation of the stress tensor at both sides of the droplet interface where $E_{n}\equiv E_{r}$ and $E_{t}\equiv E_{\theta }$, where ${\left\|ℛ\right\|}_{12}$ denotes the jump of the property, “${ℛ}_{2}-{ℛ}_{1}$” across the interface.

(21) $${\left\|T_{M,\theta }\right\|}_{12}=\frac{9}{2}\frac{S-R}{{\left(R+2\right)}^{2}}\varepsilon_{2}E_{o}^{2}\sin2\theta ,$$

(22) $${\left\|T_{M,r}\right\|}_{12}=\frac{9}{2}\frac{\left(R^{2}+1-2S\right)\cos^{2}\theta +S-1}{{\left(R+2\right)}^{2}}\varepsilon_{2}E_{o}^{2}.$$

From these equations, one can deduce the direction of shear stresses that are particularly important in determining the flow circulations. When R>S, the direction of net shear stress is from the equator to the poles and vice versa.

#### 3.2.2. Fluid Flow Equations and Their Solutions

The governing equation for fluid motion with velocity ($\vec{u})$, viscosity (μ), and pressure (p) is the conservation of mass and momentum.

(23) $$\nabla \cdot \vec{u}=0,$$

(24) $$\mu \nabla^{2}\vec{u}+f_{E}=\nabla p.$$

The divergence of the Maxwell stress tensor from Equation (20) gives:

(25) $$f_{E}=\nabla \cdot T_{M}=Q\vec{E}-\frac{1}{2}{\vec{E}}^{2}\nabla \varepsilon .$$

As discussed above, if the charge instantly accumulates at the interface, for example, for a leaky dielectric system, the volumetric charge density (Q) is zero in the bulk and for liquids with constant properties, ∇ε=0. Thus, $f_{E}=0$ in the bulk and Equation (24) reads

(26) $$\mu \nabla^{2}\vec{u}=\nabla p.$$

This shows that the electric force affects the solution only through the momentum jump boundary condition at the interface. The solution of the resulting Stokes equation can be obtained by introducing a stream function at the two sides of the droplet interface [100].

(27) $$u_{r}=\frac{1}{r^{2}\sin\theta }\frac{\partial \psi }{\partial \theta },$$

(28) $$u_{\theta }=\frac{1}{r\sin\theta }\frac{\partial \psi }{\partial r},$$

(29) $$D^{4}\psi =0.$$

$D^{4}=D^{2}\left(D^{2}\right)$, where $D^{2}=\partial^{2}/\partial r^{2}+\left(\sin\theta /r^{2}\right)\left(\partial /\partial \theta \right)\left[\left(1/\sin\theta \right)\left(\partial /\partial \theta \right)\right]$ is a well-known differential operator appearing in Stokes equation. The general solution of ψ is made specific by the following boundary conditions:

(30) $$u_{r,1}\;\mathrm{and}\;u_{\theta ,1}\;\mathrm{should}\;\mathrm{be}\;\mathrm{bounded}\;\mathrm{at}\;r=0,\;u_{\theta ,1}\left(a,\theta \right)=u_{\theta ,2}\left(a,\theta \right),\;u_{r,1}\left(a,\theta \right)=u_{r,2}\left(a,\theta \right)=0,\;{\left\|{\vec{T}}_{H,\theta }\right\|}_{12}+{\left\|{\vec{T}}_{M,\theta }\right\|}_{12}=0,\;u_{r,2}\left(\infty ,\theta \right)=u_{\theta ,2}\left(\infty ,\theta \right)=0.$$

The solution using the above boundary conditions at a steady state gives:

(31) $$\psi_{1}=\left[{\left(\frac{r}{a}\right)}^{3}-{\left(\frac{r}{a}\right)}^{5}\right]U_{max}a^{2}\sin^{2}\theta \cos\theta ,$$

(32) $$\psi_{2}=\left[{\left(\frac{a}{r}\right)}^{2}-1\right]U_{max}a^{2}\sin^{2}\theta \cos\theta ,$$

(33) $$U_{max}=\frac{9\varepsilon_{2}E_{o}^{2}a\left(S-R\right)}{10\mu_{2}\left(1+M\right){\left(R+2\right)}^{2}}$$

The velocity field is obtained from Equations (27) and (28).

(34) $$u_{r,1}=\left[{\left(\frac{r}{a}\right)}^{3}-\left(\frac{r}{a}\right)\right]U_{max}\left(1-3\cos^{2}\theta \right),$$

(35) $$u_{\theta ,1}=\frac{1}{2}\left[5{\left(\frac{r}{a}\right)}^{3}-3\left(\frac{r}{a}\right)\right]U_{\max}\sin2\theta ,$$

(36) $$u_{r,2}=\left[{\left(\frac{a}{r}\right)}^{2}-{\left(\frac{a}{r}\right)}^{4}\right]U_{max}\left(1-3\cos^{2}\theta \right),$$

(37) $$u_{\theta ,2}={\left(\frac{r}{a}\right)}^{4}U_{\max}\sin2\theta .$$

From these equations, it is evident that the flow direction is from the poles to the equator if $U_{max}$ is positive and vice versa. On the other hand, the sign of $U_{max}$ depends on the sign of quantity (S−R).

#### 3.2.3. Taylor Deformation Parameter (D)

The deformation or distortion of the droplet interface is caused by the electric and the hydrodynamic fields being discontinuous at the interface, resulting in the interfacial stress jumps countered by the interfacial capillary stresses.

(38) $$\left[{\left\|\sigma_{H,r}\right\|}_{12}+{\left\|T_{M,r}\right\|}_{12}\right]=\gamma \kappa ,$$

where $\sigma_{H,r}$ are the normal hydrodynamic stresses (${\left\|\sigma_{H,r}\right\|}_{12}={\left\|T_{H,r}\right\|}_{12}-{\left\|p\right\|}_{12}$). ${\left\|T_{H,r}\right\|}_{12}$ is found from the velocity field and ${\left\|p\right\|}_{12}$ is obtained by integrating Equation (26). The coupling between hydrodynamic and electric fields occur at the interface.

For small deformations:

(39) $$\kappa =\frac{2}{a}+\frac{8D}{3a}\left(3\cos^{2}\theta -1\right),$$

where the Taylor’s deformation parameter D is defined as:

(40) D=(L−W)/(L+W),

where L and W represent the length of the axis of the deformed droplet parallel and perpendicular to the electric field direction, respectively. Additionally:

(41) $${\left\|\sigma_{H,r}\right\|}_{12}=\left(2+3M\right)\left(1-3\cos^{2}\theta \right)\frac{\mu_{2}U_{max}}{a}.$$

**Figure 2:**
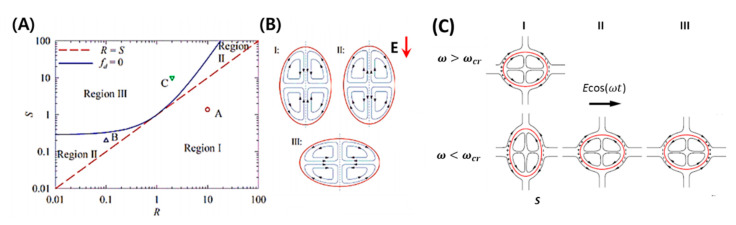
Steady deformation-circulation maps of single-phase emulsion droplet under weak DC/AC electric field. (**A**) Deformation regimes depending on conductivity ratio (*R*), permittivity ratio (*S*), and viscosity ratio (*M*) as predicted by leaky dielectric theory (LDM). (**B**) Flow patterns surrounding the droplet in DC electric field for different regimes as mentioned in (**A**) [68]. (**C**) Flow patterns surrounding the droplet in an AC electric field for different regimes [101].

Substitution from Equations (22), (39) and (41) in Equation (38) gives a deformation of droplet in a uniform electric field:

(42) $$D_{DC}=\;\frac{9}{16}Ca_{E}f_{d},\;f_{d}=\frac{1}{{\left(R+2\right)}^{2}}\left[\left(R^{2}+1\right)-2S+3\left(R-S\right)\left(\frac{3M+2}{5M+5}\right)\right]$$

where $Ca_{E}$ is the electric capillary number and $f_{d}$ is the deformation characteristic function When$\;f_{d}>0$, the droplet shows prolate deformation and, when $f_{d}<0$, it shows oblate deformation. A leaky dielectric droplet remains spherical if $f_{d}=0$ (See Figure 2A). The deformation-circulation map depends on *R* and *S* of the system. If S/R<1, the flow direction is from equator to pole and resulting shape of the droplet is always prolate (Region I). On the other hand, if S/R>1, the flow direction is from the pole to equator and droplet shape can be prolate (Region II) or oblate (Region III) as shown in Figure 2B.

Ajayi [3] used a domain perturbation method to improve the deformation predicted from Equation (42) by incorporating second-order function of $Ca_{E}$, and the formulations are given as [59],

(43) $$D_{DC}=k_{1}Ca_{E}+k_{2}Ca_{E}^{2}+O\left(Ca_{E}^{3}\right),$$

where

(44) $$k_{1}=\left(9/16\right)f_{d},\;k_{2}=k_{1}\left[\left(\frac{9}{5}\frac{R-1}{R+2}-\frac{1}{16}\right)f_{d}+\frac{R-S}{{\left(R+2\right)}^{2}}\beta \right],\;\beta =\frac{23}{20}-\frac{139}{210}\left(\frac{1-M}{1+M}\right)-\frac{27}{700}{\left(\frac{1-M}{1+M}\right)}^{2}.$$

The transient deformation, $D_{DC}\left(t\right)\;$of the droplet has an exponential form given as [5]:

(45) $$D_{DC}\left(t\right)=D_{\infty }\left[1-\exp\left(-t/\tau \right)\right],\;\tau =\frac{\left(19M+16\right)\left(2M+3\right)a\mu_{2}}{\left(40M+4\right)\gamma }.$$

Here, $D_{\infty }$ is the steady state deformation as calculated from Equations (42) or (43).

#### 3.2.4. Effect of AC Electric Field

The theoretical foundation was established by Torza et al. [7] who considered a droplet under an AC electric field, $E_{ext}\left(t\right)=E_{o}\cos\omega t$, within the Stokes flow regime, where ω is the frequency of applied electric field. The net normal ${\left\|T_{M,r}\right\|}_{12}$ and tangential electric stresses ${\left\|T_{M,\theta }\right\|}_{12}$, the velocity field$\;\vec{u}$, and the deformation D have a time-independent steady component (s) that is a function of frequency in addition to another important parameter and a time-dependent component (t) with frequency twice that of frequency of an applied electric field.

(46) $${ℛ}_{AC}={ℛ}_{s}+{ℛ}_{t}.$$

Here ℛ represents one of ${\left\|T_{M,r}\right\|}_{12}$, ${\left\|T_{M,\theta }\right\|}_{12}$, $\vec{u}$, and D. For the steady component of the stress, the deformation is given by an equation similar to (42), namely:

(47) $$D_{s}=\;\frac{9}{16}Ca_{E}f_{s},\;f_{s}=1-\frac{R\left(11M+14\right)+15\left(M+1\right)+S\left(19M+16\right)+15\Pi^{2}\left(M+1\right)\left(2S+1\right)}{5\left(M+1\right)\left[{\left(R+2\right)}^{2}+\Pi^{2}{\left(S+2\right)}^{2}\right]},$$

where $f_{s}$ is the deformation characteristic function for an AC electric field, which determines the sense of drop deformation, and $\Pi =\varepsilon_{2}\omega /\sigma_{2}\equiv t_{c}/t_{e}$, where $t_{c}=\varepsilon_{2}/\sigma_{2}$ and $t_{e}=1/\omega$ are the charge relaxation and the electric field time scales. For ω→0, $f_{d}=f_{s}$ and the steady deformation $\left(D_{s}\right)\;$are same as the deformation under a DC electric field, provided that

(48) $$E_{rms}=\underset{T\to \infty }{\lim}\sqrt{\frac{1}{2T}\int_{-T}^{T}{\left(E_{o}\cos\omega t\right)}^{2}dt}=\frac{E_{o}}{\sqrt{2}}\;,$$

in place of $E_{o}$ in the Taylor’s solution ($D_{s}=\left(1/2\right)D_{DC}$). For ω→∞, the steady deformation $\left(D_{s}\right)$ is same as the solution provided by Allan and Mason [97] ($D_{DC\_PD}=\left(9/16\right)Ca_{E}{\left(S-1\right)}^{2}/{\left(S+2\right)}^{2}$) for a perfect dielectric in a DC electric field, provided that $E_{rms}=E_{o}/\sqrt{2}$ is used ($D_{s}=\left(1/2\right)D_{DC\_PD}$). In this case, the charge relaxation time scale ($t_{c})$ is much larger than the timescale of the electric field ($t_{e})$ and hence the system behaves as a perfect dielectric. The transition between prolate and oblate ellipse takes place at a critical frequency defined as

(49) $$\omega_{cr}=\frac{\sigma_{2}}{\varepsilon_{2}}\sqrt{\frac{5\left(M+1\right)\left(R^{2}+1\right)+\left(9M+6\right)R+\left(16+19M\right)S\;}{5\left(M+1\right){\left(S-1\right)}^{2}}}\;.$$

For $\omega >\omega_{cr},$ $D_{s}>0$ (prolate deformation), and for $\omega <\omega_{cr}$, $D_{s}>0$, or $D_{s}<0$ (oblate deformation) (See Figure 2C). At $\omega =\omega_{cr}$, the drop remains spherical ($D_{s}=0$). For the time-dependent part,

(50) $$D_{t}=\frac{9}{32}Ca_{E}\left[\frac{\Phi \cos\left(2\omega t+\alpha \right)}{{\left(1+k^{2}\lambda_{2}^{2}\right)}^{1/2}}\right],$$

where

(51) $$\Phi \equiv {\left\{f_{s}^{2}+\frac{\Pi^{2}\left(19M+16\right){\left(R-S\right)}^{2}\left[\left(M+1\right)\left(20S-1\right)-3\right]}{25{\left(M+1\right)}^{2}{\left[R^{2}{\left(1+2R\right)}^{2}+\Pi^{2}{\left(S+2\right)}^{2}\right]}^{2}}\right\}}^{1/2},\;k\equiv \omega \mu_{2}a/\gamma ,\;\cos\alpha \equiv \frac{h^{*}+\bar{h^{*}}}{2I},\;\sin\alpha \equiv \frac{h^{*}-\bar{h^{*}}}{2I𝕚},\;h^{*}\equiv \frac{F_{r}^{*}-\lambda_{1}F_{\theta }^{*}}{1+𝕚k\lambda_{2}},\;F_{r}^{*}\equiv A^{2}\left(5-2S\right)-2A+1,\;F_{\theta }^{*}\equiv \frac{R\left(1+𝕚\Pi \right)}{{\left[R+2+𝕚\Pi \left(S+2\right)\right]}^{2}},\;\lambda_{1}\equiv \frac{3\left(3M+2\right)}{5\left(M+1\right)},\;\lambda_{2}\equiv \frac{\left(19M+16\right)\left(2M+3\right)}{20\left(M+1\right)},\;A\equiv \frac{1+𝕚\Pi }{2+R+𝕚\Pi \left(S+2\right)},$$

where 𝕚 is imaginary unit, and $\bar{h^{*}}$ represents the complex conjugate of $h^{*}$.

To evaluate how much oscillatory deformation ($D_{t}$) affects the overall deformation compared to the steady deformation ($D_{s}$), we consider the following ratio:

(52) $$\frac{D_{t}}{D_{s}}=\frac{\Phi \cos\left(2\omega t+\alpha \right)}{2f_{s}{\left(1+k^{2}\lambda_{2}^{2}\right)}^{1/2}},$$

Obtained from Equations (47) and (50). The dimensionless parameter k, defined by Equation (51) and which plays an important part in characterizing the ratio ($D_{t}/D_{s}$), may be considered as the ratio of the oscillatory hydrodynamic stress ($\omega \mu_{2}$) and the capillary pressure (γ/a) at the droplet interface. When $\omega \mu_{2}\to \infty$ and γ/a remains fixed, k→∞ and $D_{t}/D_{s}\to \infty$, the droplet surface being unable to respond to the oscillating stress.

## 4. Single-phase Emulsion Droplets

### 4.1. Deformation

As discussed in Section 3, the droplet can undergo prolate or oblate steady deformation. The EHD of single-phase emulsion droplets can also be categorized with respect to electric capillary number ($Ca_{E}$) and steady or transient deformation (*D*). Taylor’s LDM predicts the deformation of droplets within low-deformation limit, i.e., when $Ca_{E}\ll 1$ adequately well. In this case, the droplet adopts a spheroidal shape. If the electric field strength is increased, the droplet is susceptible to instabilities and breakups, and LDM no longer remains valid. The steady and transient deformation of droplets at a low electric field is vastly studied and is well understood. In literature, various analytical, numerical, and experimental studies addressing different issues, such as effect of inertia, charge convection, charge relaxation, and type of electric field (AC or DC, uniform or Non-uniform), are found [4,5,6,8,9,11,24,54,61,68,102,103,104,105,106,107,108,109,110,111].

#### 4.1.1. Under DC Electric Field

**Figure 3:**
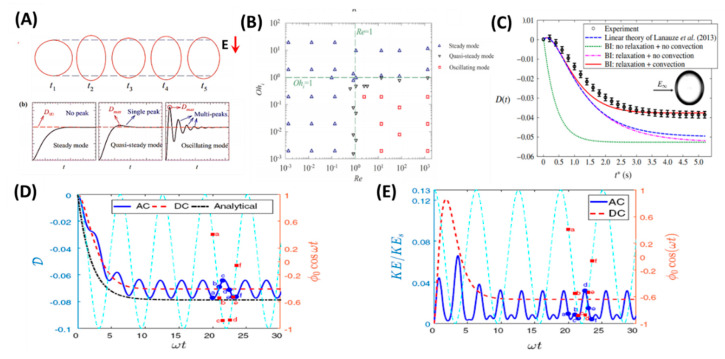
Transient deformation of single-phase emulsion droplet under a weak DC/AC electric field. (**A**) Three different modes of deformation encountered in transient deformation of a droplet. (**B**) The mapping of deformation modes as mentioned in (**A**) on Ohnesorge number (*Oh*) vs. Reynolds Number (*Re*) parametric space [68]. (**C**) The effect of charge relaxation and convection on transient deformation of a silicone oil droplet in castor oil [60]. (**D**) Evolution of the deformation with time. (**E**) Evolution of the kinetic energy of the fluid system with time [101].

The transient deformation of a droplet essentially depends on two dimensionless numbers, Ohnesorge number (*Oh*) and the Reynolds number (*Re*) [68]. When Re≪1 or Oh>1, the droplet always has a steady mode represented by monotonic deformation during its transient evolution. Whereas, if Re≫1 and Oh<1, the droplet has an oscillating deformation before achieving the steady configuration. The droplet has a quasi-steady mode of deformation at the borderline region that distinguishes the monotonic and oscillating deformation regions (See Figure 3A,B). Feng and Scott showed that the droplet shape may change from oblate to prolate due to the inertial effects associated with the increase in the strength of the electric field [6]. The main effect of charge convection is to reduce the interfacial velocity that results in more deformation of a prolate droplet, while an oblate droplet undergoes lesser deformation. Lanauza [103] and Das et al. [4] showed that the surface charge convection causes formation of “charge shocks” near an oblate droplet’s equatorial region. The experimental data for the deformation of a silicone oil droplet in castor oil is as shown in Figure 3C. All the theoretical or numerical models fail to capture the dynamics except the one that considers the charge relaxation and convection effects.

#### 4.1.2. Under AC Electric Field

Although the sense of steady-state flow circulation pattern in an AC electric field is the same as that in a DC electric field, the sense of $D_{s}$ is not the same as that of $D_{DC}$. Instead, it depends on the field frequency ω being below or above a critical frequency $\omega_{c}$, defined by Equation (49) [7,101,112]. Esmaeeli and Halim [101] compared the transient deformations of droplet in a DC electric field to those in an AC electric field using direct numerical simulation (DNS). For the range of the physical parameters used in their study, the evolution of the drop deformation $D_{\mathrm{AC}}$ with time t showed that the drop settled to its quasi-steady state in a relaxation time $t_{r}=\left(\mu_{1}+\mu_{2}\right)a/\gamma$ (See Figure 3D). Additionally, they showed that the kinetic energy of the fluid system is minimized when the rate of the deformation ($\partial D_{AC}/\partial t$) is zero, where there is a state of minimum or maximum deformation. On the other hand, it is maximized when in the state of steady deformation during the deformation half-cycle (See Figure 3E). Esmaeeli [112] showed flow field in and outside of drop in an AC electric field, and evolution of the total deformation $D_{AC}$ versus nondimensional time ωt for three different deformation regions.

### 4.2. Instabilities and Breakups

The dynamics of a droplet under a strong electric field when $Ca_{E}≳1$ depends on the ratio of electrical conductivity to electrical permittivity (R/S).

#### 4.2.1. Prolate Deforming Droplets

**Figure 4:**
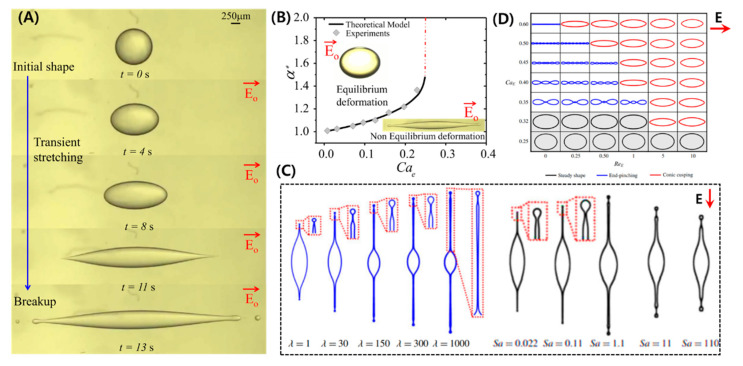
Breakup of a prolate droplet under a strong DC electric field. (**A**) Transient evolution of a castor droplet in silicone oil and breakup at the side ends. (**B**) Non-dimensional stretching vs. electric capillary number leading to instability (reproduced from [15] with permission from The Royal Society of Chemistry). (**C**) Effects of viscosity ratio and liquid conductivity (in terms of Saville number) on the droplet shapes [114]. (**D**) Droplet breakup mode transition depending on electric Reynolds Number [115].

In most of the studies related to single-phase droplets, the droplet generally settles to an equilibrium shape under a relatively weak electric field. However, if the strength of the electric field is high and R/S>1, then the droplet becomes unstable and thereby disintegrates or breaks into daughter droplets. This topic is covered by different authors who have tried to determine the critical parameters to distinguish between the stable and unstable regimes [6,8,9,15,22,26,59,61,113]. The numerical models are developed assuming ellipsoidal or slender shapes. Sherwood [61] studied the droplets under a strong electric field using boundary integral method. Similar to Sherwood, Lac and Homsy [59] used an axisymmetric boundary integral method and studied droplet stability by changing different parameters. Bentenitis and Krause [8] theoretically studied the large deformation of the droplets, extending the conventional leaky dielectric model by analytical solutions of an electric field and flow field in a framework of spheroidal deformation. Figure 4A shows a transient evolution of a droplet that continuously stretches under the applied electric field and eventually becomes unstable, ejecting daughter droplets at the side apex. It was reported that as the droplet’s semi-major axis becomes greater than 1.5 times its undeformed radius, the droplet becomes unstable [15]. For the case of a castor oil droplet in silicone oil, this instability occurred at $Ca_{E}\sim 0.25$ (See Figure 4B). However, the critical electric capillary number for a conducting droplet, such as a water droplet in silicone oil, is well known to take place at $Ca_{E}\sim 0.2\pm 0.2$ [6,61].

Two important modes of breakups are reported, such as end pinching and tip streaming [114,115]. In the former mode, the droplet is converted into bulbous-shaped lobes that eventually disintegrate, whereas in the latter case, the droplet develops sharp cone-like tips ejecting jets. These jets eventually disintegrate into tiny droplets. The transition between these modes is rather complex and depends on various parameters, such as droplet conductivity (R), viscosity ratio (M) between the liquids, and applied electric field ($E_{0}$). Figure 4C shows the effect of viscosity ratio and droplet conductivity. At M=1, the droplet forms small daughter droplets at the tip with small protrusion. As the M increases, the jet length and daughter droplet size increases. This shows the importance of viscous stresses to balance the electrical tangential stresses to form a jet. Droplet conductivity effect is represented in terms of Sa ($Sa\propto 1/\sigma_{2}$). A high-conductivity droplet ejects thinner jets and smaller daughter droplets via tip streaming. For a low-conducting droplet, the jet vanishes, and the breakup is via end-pinching due to rapid domination of capillary stresses. It is also notable that with a decrease in droplet conductivity, the jet length first increases, reaches a maximum (Sa≈1), and then it decreases [114]. It is also reported that a change of mode can also take place due to surface charge convection effects represented by $Re_{E}$, as shown in Figure 4D [115].

#### 4.2.2. Oblate Deforming Droplets

**Figure 5:**
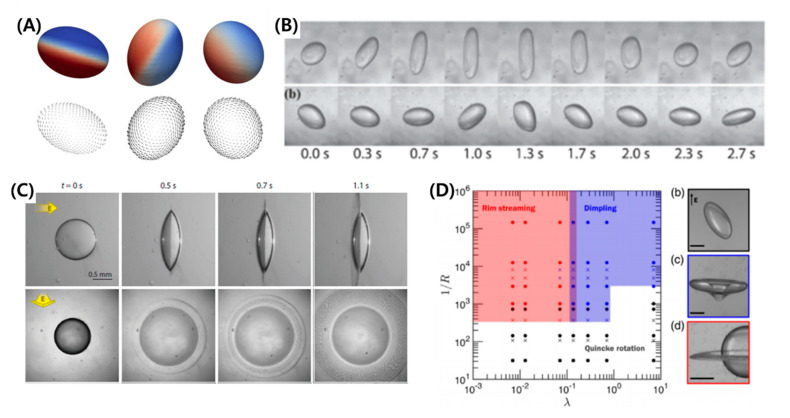
Instabilities of an oblate droplet under a strong electric field. (**A**) Electro-rotation of a droplet above the threshold electric field of Quincke effect [90]. (**B**) Irregular rotational motions [118]. (**C**) Equatorial streaming. (**D**) Mapping of different instabilities, such as electro-rotation, dimpling, and equatorial streaming [18].

The droplet undergoes electro-rotation under a strong electric field if R/S<1 due to the surface charge convection effects (See Figure 5A) [18,90,116,117,118,119]. The phenomenon is similar to the rotation of solid particles in fluid known as Quincke rotation, where the induced dipole tries to align itself with the electric field, and the resulting torque causes a rotation perpendicular to the direction of the applied electric field. It takes place when the applied electric field is above the certain threshold value ($E_{q}$). The value for a rigid sphere is given as [118,120]:

(53) $$E_{q}=\sqrt{\frac{2\mu_{2}\sigma_{2}{\left(R+2\right)}^{2}}{3\varepsilon_{1}\varepsilon_{2}\left(1-R/S\right)}}.$$

The equation is obtained by the balancing of electrical and viscous torques and is independent of the size of sphere. The equation is often used to estimate the threshold of electro-rotation for the liquid droplets and comparison with experimental results shows a relatively good approximation [117]. Results show that the droplet attains a steady tilt relative to the applied electric field and can undergo irregular rotational motions (See Figure 5B). The electro-rotation stabilizes the droplet against breaking by suppressing the droplet deformation. Recently, Brosseau et al. [18] observed very interesting phenomena, such as droplet dimpling and equatorial streaming (Figure 5C,D). In the dimpling mode, the droplet forms a torus shape; the torus subsequently breaks into relatively larger drops. This takes place for droplets with M≳1 and $Ca_{E}\sim O\left(1\right)$. In the equatorial streaming mode, the droplet flattens with the sharp edges. Concentric waves in the form of rings emanate from the thin edges that break into tiny droplets. The critical conditions for this mode are R≪1, $Ca_{E}\gg 1$ and M≲0.1.

## 5. Particle-Covered Droplets

### 5.1. Deformation

**Figure 6:**
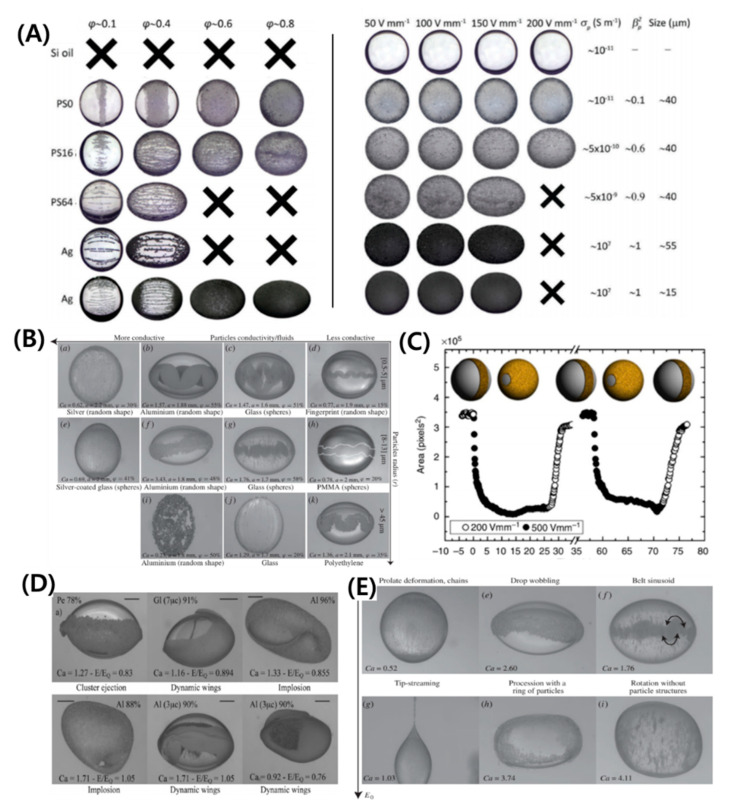
Self-assembly of particles on the droplet interface by electro-hydrodynamic flows. Under a weak electric field: (**A**) effect of particle electric conductivity and concentration on the particle structure (reproduced from [134] with permission from The Royal Society of Chemistry). (**B**) Effect of particle electric conductivity and size on the particle structure [91]. (**C**) Opening and closing of belt [21]. Under a high electric field: (**D**,**E**) effect of particle size, concentration, and electric conductivity on the particle structure [91].

A colloidal particle or Pickering droplet is formed when the particle-laden droplet or suspension, for which R/S<1, is subjected to an electric field [21,91,92,121,122,123,124,125,126,127,128,129,130,131,132,133,134,135]. Particles can be trapped at the interface more rapidly under a weak electric field (~ 0.1 kV/mm) and once trapped, they remain there. Depending on the strength of the electric field, particle concentration, electric conductivity, and size, various particle assemblies can be formed at the interface. Mikkelsen et al. [134] used low to high particle coverage (~0.1 to ~0.8), and analyzed the conductive and non-conductive particle conditions, as shown in Figure 6A. For high particle concentration of non-conducting particles, such as polystyrene under a weak electric field, the oblate deformation of silicone oil increased as more charges accumulated at the droplet surface and the EHD flows were suppressed. On the other hand, as the electrical conductivity of particles entrapped at the silicone droplet interface increased, the oblate deformation of the silicone oil droplet changed to prolate deformation. In this case, the dipole moment becomes aligned with the electric field, unlike the case of the silicone droplet with the clean interface or covered with non-conducting particles. For low particle concentrations in a range from ~0.1 to ~0.5 under a weak electric field, the particles at the interface can produce different assemblies, such as “belts” (low-conductivity particles) or “chains” (high-conductivity particles). Belts may form a static or dynamic sinusoid with a change in particle properties and electric field strength [91,134]. High-conductivity particles with regular size, such as spheres, organize into regular chains aligned with the direction of an applied electric field, while random-size particles form random assemblies (See Figure 6B). The particle belts can open and close over the entire surface of the droplet by application and removal of the electric field, respectively [21] (See Figure 6C).

### 5.2. Instabilities and Breakups

For high particle coverage under a strong electric field, the droplet adopts peculiar drum-like shapes and may even start rotating (electro-rotation phenomenon as discussed in Section 4.1.1) or implode. The particle layer buckles, leading to various outcomes, such as ejection of particle clusters or formation of ephemeral wings [122], as shown in Figure 6D. For low particle concentrations under a strong electric field, droplets with chains (high-conductivity particles) eventually break at the side ends (tip streaming), as shown in Figure 6E. Whereas, those forming belts (low-conductivity particles), either redistribute to form counter-rotating vortices, lose their structure, or eject.

## 6. Surfactant-Laden Droplets

### 6.1. Deformation

**Figure 7:**
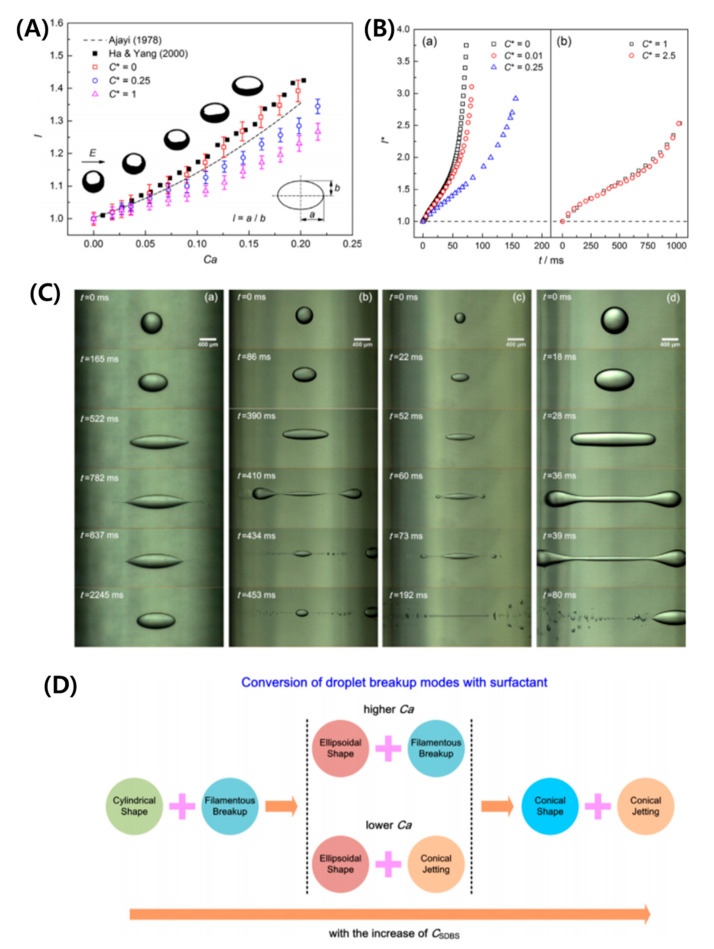
Surfactant-laden droplets under a DC electric field. (**A**) Steady deformation represented by degree of stretching (*l*) vs. electric capillary number ($Ca_{E}$) at different surfactant concentrations ($C^{*}$). (**B**) Stretching dynamics [27]. (**C**) Breakup modes. (**D**) Conversion between breakup modes [83].

The influence of interfacial properties on the droplet deformation and breakup under an electric field has also been a topic of profound interest for decades [27,71,72,73,74,75,76,77,78,79,80,81,82,83,84,85,86,87]. The surface-active molecules prompt to change in the interracial and rheological properties, leading to interfacial perturbations and breakups [23,71,136,137,138]. It is important to note that under a weak electric field, the steady deformation for a surfactant-laden aqueous droplet is lower at the same $Ca_{E}$ (calculated in terms of surfactant-free droplets), and this difference is more prominent for larger deformation [139,140] (See Figure 7A). This could be the result of the surfactant harassing the EHD flows, though the already weak flows for conducting droplets cannot fully justify it. The possible intriguing effect is the surfactant dilution with an increase in interfacial area. The larger the deformation, larger is the increased interfacial area, leading to an increased dilution and hindering the deformation significantly. The transient deformation showed an interesting result that the droplet-stretching velocity decreased with an increase in the non-dimensional surfactant concentration ($C^{*}$), however, as the surfactant concentration increased beyond critical micelle concentration ($C^{*}>1$), there was no further decrease [27] (See Figure 7B). The understanding of this dynamics is rather complex. The decrease in velocity for ($C^{*}<1$) is attributed to the re-distribution of surfactant molecules with an increase in interfacial area and non-availability of surfactant molecules from the bulk causing local surface tension gradients and Marangoni flows. These flows oppose the droplet stretching and hence the droplet stretching velocity decrease. For ($C^{*}>1$), the rate of exchange of the surfactant molecules from the bulk to the interface is still slow and saturation in terms of Marangoni effect occurs. It is noteworthy that the exchange of surfactant molecules in case of an electric field not only depends on viscosity ratio (M), but also on the permittivity (S) and conductivity (R) of the liquids, as the circulation inside and outside the droplet relies on all these parameters and can affect the droplet transient deformation.

### 6.2. Instabilities and Breakups

Unlike the surfactant-free droplet, where the breakup modes are controlled by ratios of viscosity, conductivity, and permittivity, dynamics of the interface manipulated by surfactant is another important criterion in the breakup of a surfactant-laden droplet. The breakup of a surfactant-laden aqueous droplet under a strong electric field can show different modes such as: conical shape-conical jetting, ellipsoidal shape-conical jetting, ellipsoidal shape-filamentous breakup, and cylindrical shape-filamentous breakup [83] (Figure 7C). The conversion criterion between these breakup modes with respect to electric capillary number and the increase in the surfactant concentration is specified in Figure 7D. It has been shown that this behavior is similar to the tensile-fracture process in the elastic–plastic mechanics and thus the stress-strain relationship is used to analyze the axial stretching.

## 7. Multi-Phase Emulsion Droplets

In the past few years, multi-phase droplets, such as double-emulsion droplets or compound droplets with three liquid components (core, shell, and ambient) and two interfaces (core/shell and shell/ambient), have gathered special attention due to their vast applications in various scientific areas, such as in targeted drug delivery [141], artificial supply of oxygen in blood [142], etc. The foundation of the theory of a double-emulsion droplet under an electric field is that of a single-emulsion droplet as discussed in Section 3. A more comprehensive coverage of the governing equations is not included in this review and can be found in literature [143,144]. An A/B/A type (core/shell/ambient) of a double-emulsion droplet has the same liquid in the core, and ambient, and is essentially a two-phase problem. On the other hand, an A/B/C type has three distinct liquid components. In this section, we first consider the dynamics of double-emulsion droplets or compound droplets within a weak electric field ($Ca_{E}\ll 1$) and, lately, under a strong electric field.

### 7.1. Deformation

The dynamics of a double-emulsion droplet within a weak electric field or small deformation limit is extensively reported and well understood [33,67,93,94,143,144,145,146,147,148,149,150,151]. A/B/C types of a double-emulsion droplets can show four distinct modes of deformation of core/shell-shell/ambient, i.e., prolate-prolate (PP), prolate-oblate (PO), oblate-prolate (OP), and oblate-oblate (OO), which entirely depends on the ratio (R/S) across each of the interface [67,93,143]. If R/S<1, the interface deforms oblate and if R/S>1, it deforms prolate. For an A/B/A type, only two modes of core-shell deformation, i.e., PO and OP, are possible. The use of an electric field in the centering of the core droplet can be found extensively in literature and is well understood [42,145,148,149,150]. Oguz and Sadhal [145] conducted the earliest study on the effects of a weak electric field on the dynamics of compound droplets. They showed that under an electric field, stable equilibrium configuration of a compound drop is possible, which may not be possible without using an electric field. T. Sukada et al. [147] showed that deformation and flow strength are positively related to the strength of an electric field and core-to-shell droplet volume ratio, using theoretical analysis and experimental observations.

**Figure 8:**
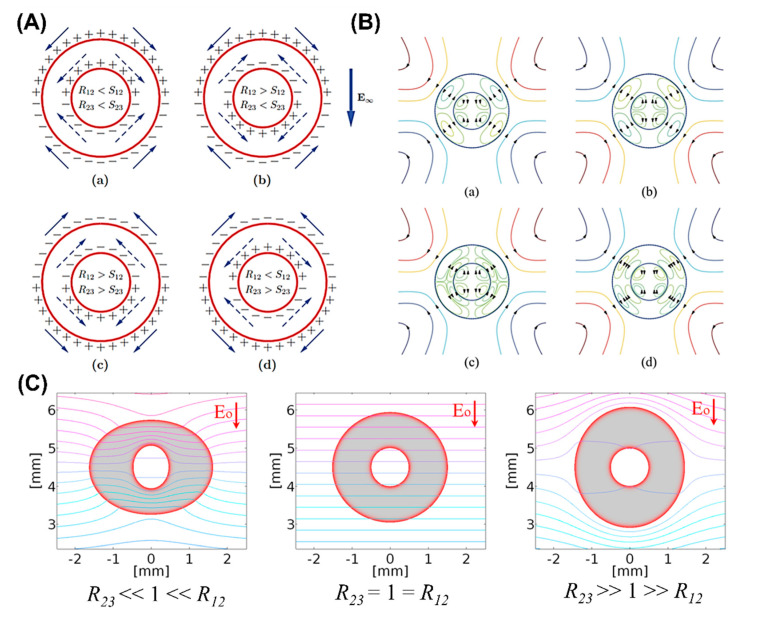
Dynamics of a double-emulsion droplet depending on relative magnitudes of permittivity ratio and conductivity ratio under a weak electric field. (**A**) Free electric charge distributions for four distinct cases. Solid and dashed arrows show the direction of tangential electric stresses at the interfaces. (**B**) Scenarios of different flow patterns [143]. (**C**) Contours of electric potential lines [14].

Behjatian and Esmaeeli [143] studied the A/B/C type concentric emulsion analytically under a DC electric field using domain perturbation method and showed various free-charge distributions surrounding the interfaces depending on the relative magnitudes of R and S (See Figure 8A). They also studied the deformation-time history of the double droplets under weak electric fields [93]. Their analysis was based on the Laplace equation of charge distribution and considering the droplets to have no net charge. Same as in LDM, electro-neutrality was assumed for the bulk liquids, and electric and hydrodynamic tractions became discontinuous at the two interfaces. For $R_{ij}<S_{ij}$, the charge sense on the upper half of the core/shell and shell/ambient interface are the same with respect to each other and as that on the supply electrode (+), while the lower half has the negative charge (−). The oblate deformation of the interfaces and the OO configuration is expected. For $R_{ij}>S_{ij}$, the charges are reversed. Here, ij=12. for core/shell and ij=23 for shell/ambient, respectively. The prolate deformation of the interfaces and the PP configuration is expected. For OP and PO configurations, the core/shell and shell/ambient interfaces have opposite charges with respect to each other. By changing the direction of electric field, the charge sense can be reversed but the direction of the tangential electric stress remains the same. Unlike the case for a single-phase emulsion droplet, it is possible to get two counter-rotating vertices in a multi-phase emulsion droplet, as shown in Figure 8B. The generation of toroidal vortices depends on the electrical properties of the constituting liquids; however, the precise criterion has still not yet been established. The extent of the deformation of the inner core droplet also depends on the electrical conductivity of the shell. Soni et al. [144] extended the analytical analysis to an AC electric field. They showed that in the limit of zero frequency, their results reduced to those derived by Behjatian and Esmaelli [143]. Abbasi et al. [14] showed that for shells comprised of fewer conductivity liquids and $R_{23}\ll 1\ll R_{12}$, the electric potential lines are densely concentrated in the shell region and reach the core/shell interface easily, causing deformation of both the core and the shell droplets. On the other hand, if shell liquid has large electric conductivity and $R_{23}\gg 1\gg R_{12}$, it behaves like a shield and electric potential lines cannot penetrate through it, resulting in a negligible core deformation (See Figure 8C). While Behjatian and Esmaeeli reported a steady droplet configuration under a weak electric field, it was later reported in an experimental study [13] that owing to electrophoretic effects and the presence of net charge arising from the existence of electric double/diffuse charge layers on the droplet, the steady configuration was not possible for some of the cases. As the charged core droplet experiences the electric field, it moves towards the droplet side apex and is eventually dispensed into the ambient.

### 7.2. Instabilities and Breakups

**Figure 9:**
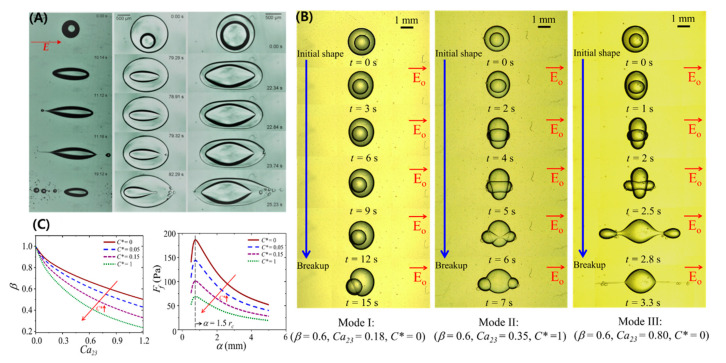
Breakups of a double-emulsion droplet under a strong electric field. (**A**) Emulsion with conducting symmetric core [95]. (**B**) Emulsion with surfactant-laden aqueous core. (**C**) Criterion for core instability and critical electric capillary number for breakups (reproduced from [16] with permission from The Royal Society of Chemistry).

This feature of multi-phase droplets is relatively less explored. Only a few studies have explored the breakups within a certain range of electric and hydrodynamic properties [13,14,16,25,30,95,152]. In one of the earliest studies, Ha and Yang established the breakups of conducting emulsion droplets comprised of inhomogeneous inner phase encapsulated by an outer membrane phase suspended in silicone oil [25]. Castor oil and aqueous phase were used to form oil-in-water (o/w) or water-in-oil (w/o) emulsions, depending on their fraction of volume, comprising either the membrane or non-uniform inner droplet phase. They recognized that the existence of non-uniformity in the form of multiple inner droplets can significantly alter the breakup modes and stability as compared to homogenous Newtonian conducting droplets. Pinch off and tip streaming modes of emulsion-breaking were observed. Soni et al. [95] explored the breaking of a A/B/C type droplet comprised of NaCl aqueous/castor oil/silicone oil in an AC electric field exhibiting PP mode of deformation, as shown in Figure 9A. The breaking of the core occurred at a larger strength of electric field compared to a NaCl aqueous droplet in a bulk castor oil. This was due to less penetration of electric potential as the shell was acting as a shield. Recently, Abbasi et al. explored the dynamics of a double-emulsion droplet with a low electrical conductivity shell beyond the small deformation limit in two separate studies and explored various breakup modes of a core droplet under varying physical and electrical parameters, such as radius ratio, electric capillary number, and/or interfacial tension [13,16]. In the former, breakups of a A/B/A type of double-emulsion comprised of castor oil/silicone oil/castor oil (core/shell/ambient) for which PO mode of deformation occurs is thoroughly explored. Four distinct breakups were reported, such as uni-directional and three different bi-directional breakups. The charge convection effects caused a change in the breakup of the core from the bulbous-shaped lobes to the formation of conical ends. The breakup modes were then plotted on a core-to-shell droplet radius ratio vs. electric capillary number parametric space. In the later study, a surfactant was introduced at the aqueous core phase of an A/B/C type of double-emulsion to change the core/shell interfacial tension [16]. The core droplet instability lead to the formation of asymmetric Janus and snowman-like stable or unstable ternary droplets, as shown in Figure 9B. By theoretical analysis, the length of stretched semi-major axis of the core at which the instability occurred was established to be 1.5 times its initial undeformed radius, and breakup modes were delineated on a radius ratio vs. electric capillary number parametric space at different non-dimensional surfactant concentrations (See Figure 9C). It is pertinent to mention here that the dynamics of a double-emulsion droplet exhibiting OO mode of deformation under a strong electric field still remains unexplored.

## 8. Applications

### 8.1. Mass Production of Micro/Nano Droplets

**Figure 10:**
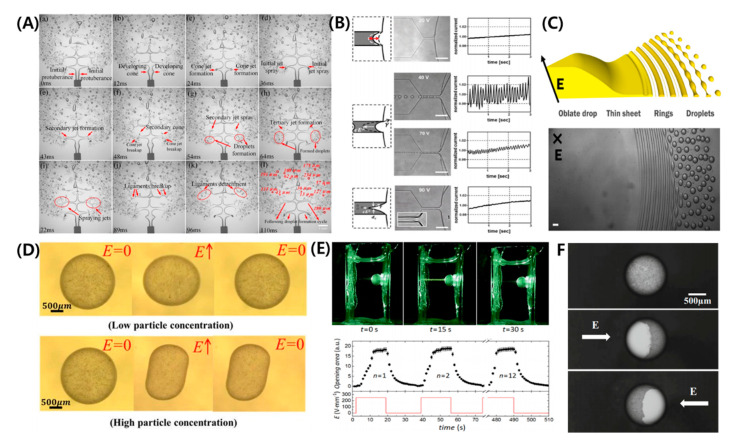
Applications of electro-hydrodynamics (EHD) of emulsion droplets. (**A**) Electric-field-induced breakup of a liquid column from a nozzle, and small droplet formation behavior [158]. (**B**) Electric production of emulsion droplets in a microfluidic system [161]. (**C**) Droplet generation via streaming from the equator of a drop in an electric field [18]. (**D**) Stabilizing the shape of deformed colloidal particles by the interfacial jamming of nanoparticles [121]. (**E**) Optical diaphragm based on the particle self-assembly [162]. (**F**) Generation of an aluminum Janus particle using an electric field [163].

EHD-based electric shear stress is used to produce tiny droplets by cutting off the liquid interface formed under the nozzle, which is called electrospray [153]. Also, in droplet-based microfluidic systems, electric fields are used to generate smaller droplets [154]. In addition to generating simple tiny single droplets or particles, these techniques can be used to make more complex droplets, such as double droplets [141,155], and thus many studies have been conducted in the fields of bioengineering [156], medical [157], and pharmaceutical [141]. Similarly, breakup at high voltage was observed in free-emulsion droplets [15] or emulsion droplets anchored on a nozzle [158,159] (Figure 10A,B). The droplet that is stretched under a strong electric field produces tiny daughter droplets at both ends, which is called tip streaming [15,25]. Using this phenomenon, it is possible to generate many small droplets ranging from sub-nano to micro size. Moreover, under special conditions, the droplets show equatorial streaming, which can be used to create a large number of micro droplets [18] (Figure 10C). In a more complex case, the condition in which a compound droplet breaks up was analyzed by Su et al., through a numerical analysis method [160].

### 8.2. Functional Droplet/Particle Synthesis

The electric field can be used to produce and manipulate various types of droplets that are not spherical. Brosseau et al. used cross-linking UV curable polymer (NOA 81, Norland Optical Adhesive, Norland Products Inc., East Windsor, NJ, USA) drop to make spheroidal shapes via EHD deformation [164]. On the other hand, in the case of droplets covered with high-concentration particles, the unique shapes deformed by the EHD flow can be fabricated due to particle jamming even if the electric field is removed [121,128,130] (Figure 10D). Manipulating particles anchored on the droplet interface is one of the promised techniques for designing functional droplet. Using active structuring of a particle-covered droplet under the electric field, Rozynek et al. demonstrated a millimeter-sized optical diaphragm [162] (Figure 10E). Li et al. redistributed aluminum nanoparticles on an o/w emulsion droplet via an electric field for generating a Janus droplet [163] (Figure 10F). Moreover, an AC electric field stabilized the double-emulsion droplet by centering the core droplet, which can be used to synthesize a polymer shell or hollow particle [42].

### 8.3. Micro-Rotor

**Figure 11:**
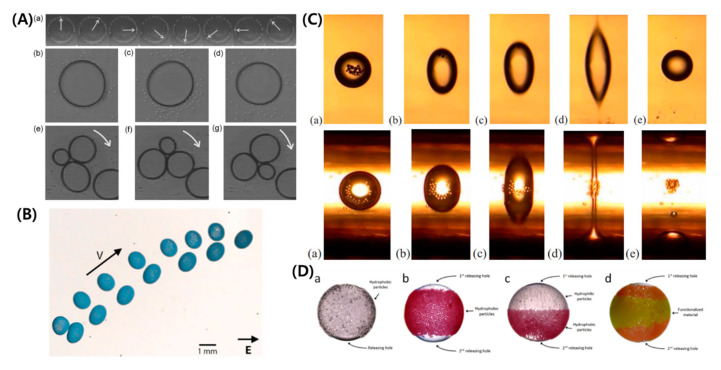
Applications of EHD of emulsion droplets. (**A**) Rotation of glass-sphere-covered air bubbles under a DC electric field [166]. (**B**) Pickering droplet propulsion [92]. (**C**) Removal or collection of particles from a w/o emulsion using an electric field [170]. (**D**) The releasing holes for material transportation from/to the capsule [171].

When R/S<1, a droplet or particle shows a unique phenomenon, called Quincke rotation, under a strong electric field. Particles on the electrode show a rolling or hovering motion with Quincke rotation [165,166]. Many theoretical or experimental researches have been conducted for Quincke rotation of polymer particles to make a rotor [165,167], motor [168], pump [169], and so on. Similarly, a single droplet with/without particles also shows electro-rotation with ellipsoidal deformation (Figure 11A). Electrohydrodynamic propulsion of two Pickering droplets placed at a close proximately takes place at different angles relative to an applied electric field with the propulsion velocities approaching 0.1 mm/s [92]. The electric field is below the threshold for the Quincke rotation and the drops may co-rotate or counter-rotate perpendicular to the field (See Figure 11B).

### 8.4. Encapsulation/Target Delivery

The double emulsion type capsulation technique is an important technique in the process of drug delivery or producing functional particles, but the resulting double emulsion may be too unstable or too stable for these purposes. However, if an electric field is used, the core droplet can be moved to the center [94,148] or ejected outward [13,14]. Tucker–Schwartz et al. showed that in the process of encapsulation of a material having a density difference with a photocurable polymer, centering through an electric field has a great influence on the stability of the capsule after the curing process [42]. Jia et al. used an AC field to initiate the release process and control the release direction of actives encapsulated in single- and dual-core double-emulsion droplets [172]. Deng et al. demonstrated a two-step selective controlled release under an AC field based on the discrepancy of the shell thickness or core conductivity of the encapsulated droplet [173]. Moreover, some studies showed that the electric field can be used to manipulate the particles inside the droplet or on the droplet interface. Based on this techniques, Nudurupati et al. demonstrated that it was able to extract particles inside the droplet [170] (Figure 11C). Rozynek et al. suggested that the release rate of some areas of the droplet, named releasing holes, can be increased by rearranging the particles using an electric field [171] (Figure 11D).

## 9. Conclusions and Future Perspectives

In this review, we discussed the droplet EHD in detail. We discussed the features such as droplet deformation, instabilities, and breakups for single-phase and multi-phase droplets. The single-phase droplet deforms prolate or oblate and undergoes instabilities above a threshold value of the electric field. The low limit deformation is well-predicted by so-called Taylor’s leaky dielectric model, which is the most established theoretical model to date for the droplet electrohydrodynamics, neglects the effects of diffuse charge layers near the interfaces, and thus is incomplete to explain the electro-migration of droplets under a direct current electric field. Therefore, an effort to derive a complete model incorporating the electro-kinetic theory for liquid-liquid interface is required. Interesting phenomena, such as tip streaming or equatorial streaming, can be used to produce many tiny droplets. Particles at the emulsion interface can be manipulated to produce intriguing assemblies, such as belts, chains, and caps, by controlling the physical and electrical parameters. High particle concentration can produce colloids undergoing rotating flows under a strong electric field. Use of surfactant molecules alter the deformation and droplet breakup dynamics. Development of simulation models are required to understand the electrohydrodynamics of more complex interfaces, and those laden with surfactant or coated with particles merit further investigations. A double-emulsion droplet shows different breakups and can be controlled to produce Janus and ternary droplets. Simulating three-phase emulsion droplets under an electric field is likely to display further interesting dynamics.

The applications of droplet EHD can be broad, such as mass production of micro droplets, functional particle synthesis, development of micro-rotors, rigid ellipsoids, or complex shapes, and so on. Such a method could then be implemented on a microfluidic platform, if a large quantity of rigid, deformed complex shapes is to be produced. However, unfortunately the demonstration of droplet EHD on a mass scale to produce species or development of new products has still not been fully demonstrated. Still, there remain many issues and challenges that are either less explored or not explored at all and warrant further investigations.

## Figures and Tables

**Table 1:** Summary of the most prominent works related to droplet electrohydrodynamics.

Ref.	Approach ^a^	Method ^b,c^	$\mathrm{Electric}\;\mathrm{Capillary}\;\mathrm{Number}\;(Ca_{E})$	Deformation Type	Characteristics	Remarks
Single-phase droplet (clean)						
[1]	E and A	Leaky-dielectric model, o/o emulsion (S/C and C/S),	$Ca_{E}<1$	Both	Deformation	DC field
[7]	E and A	Leaky-dielectric model, o/o emulsion (S/C, C/S, etc.) w/o emulsion (W/C, W/S, etc.)	$Ca_{E}<1$	Both	Deformation, pinch-off, dimpling	DC and AC field
[18]	E and A	Leaky-dielectric model, o/o emulsion (S/C),electrolyte (AOT, TBAB), linear stability analysis	$Ca_{E}\approx 4$	Oblate (*R/S* > 1)	Streaming from equator, Quincke rotation, dimpling	DC field, micro-droplet generation
[24]	E	Leaky-dielectric model, w/o emulsion (W/S, G/S, etc.), o/o emulsion (C/S), non-Newtonian emulsion (PAM and XAN solution)	$Ca_{E}<1$	Prolate (*R/S* < 1)	Deformation, pinch-off, tip streaming	DC field, effect of viscosity
[90]	A and N	Leaky-dielectric model, boundary element method, interfacial charge convection	$Ca_{E}<3$	Oblate (*R/S* > 1)	Deformation, Quincke rotation	DC field, 3D simulation
Single-phase droplet (surfactant-laden)						
[72]	E and A	Leaky-dielectric model, linear stability analysis, o/o emulsion (PVDF/PS, S/C, C/S), w/o emulsion (W/S), surfactant (PS-b-PMMA, Tween 60, etc.), non-Newtonian fluid (XAN solution)	$Ca_{E}<1$	Both	Deformation, bulbous end, tip streaming	DC field, effect of viscosity, non-ionic surfactant
[84]	E and N	Level-set method, ghost-fluid method, w/o emulsion (W/Marcol 52), surfactant (Span 80)	$Ca_{E}$ < 0.3	Prolate (R/S < 1)	Deformation, breakup	DC field, non-ionic surfactant
Single-phase droplet (particle-covered)						
[91]	E and A	Leaky-dielectric model, o/o emulsion (S/C), particle (Al, glass, PMMA, PE, etc.)	$Ca_{E}<5$	Oblate (*R/S* > 1)	Deformation, Quincke rotation	DC field, particle self-assembly
[92]	E and A	Leaky-dielectric model, o/o emulsion (S/C), particle (PE)	1 < $Ca_{E}$ < 8	Oblate (*R/S* > 1)	Quincke rotation	DC field, micro-motor
Multi-phase droplet (clean)						
[13]	E, A, and N	Leaky dielectric model, level-set method, o/o/o emulsion (C/S/C), electrolyte (Red-O dye)	$Ca_{E}\leq 1$	Prolate/Oblate	Deformation, pinch-off, bulbous end, tip streaming	DC field, effect of viscosity, and R/S
[93]	A	Leaky-dielectric model, closed form analytical solution, o/o/o emulsion (C/S/C, C/Corn/S, S/C/S, etc.)	$Ca_{E}$ < 0.1	Both/Both	Deformation	DC field
[94]	E	o/w/o emulsion (S/W/S), w/o/w/o emulsion (W/S/W/S),	$Ca_{E}$ < 0.1	Oblate/Prolate, P/O/P	Eccentricity stability, electro-phoresis	DC field
[95]	E and N	Leaky dielectric model, phase field model, w/o/o emulsion (W/C/S)	$Ca_{E}<1$	Prolate/Prolate	Deformation, pinch-off, tip streaming	AC field
Multi-phase droplet (surfactant-laden)						
[16]	E and A	Leaky dielectric model, w/o/o emulsion (W/S/C), surfactant (Tween 80)	$Ca_{E}\leq 1$	Prolate/Oblate	Deformation, bulbous end	DC field
[25]	E and A	Leaky dielectric model, o/w/o emulsion (HC/WE/S), w/o/o emulsion (WE/C/S), surfactant (Span85, Brij58)	$Ca_{E}<1$	Both/Oblate	Pinch-off, tip streaming	DC field, multi-core double emulsion

^a^ E: Experimental, A: Analytical, N: Numerical. ^b^ S: Silicone oil, C: Castor oil, W: DI water, G: Glycerol solution, HC: Hexane and chlorobenzene, WE: Water-ethyl alcohol mixture. ^c^ PAM: Polyacrylamide, XAN: Xanthan gum, AOT: Dioctyl sulfosuccinate sodium salt, TBAB: Tetrabutylammonium bromide.

## References

[1] Taylor G. (1966). Studies in electrohydrodynamics. I. The circulation produced in a drop by electrical field. Proc. R. Soc. Lond. Ser. A Math. Phys. Sci..

[2] Melcher J.R., Taylor G.I. (1969). Electrohydrodynamics: A Review of the Role of Interfacial Shear Stresses. Annu. Rev. Fluid Mech..

[3] Ajayi O. (1978). A note on Taylor’s electrohydrodynamic theory. Proc. R. Soc. Lond. Ser. A Math. Phys. Sci..

[4] Das D., Saintillan D. (2017). A nonlinear small-deformation theory for transient droplet electrohydrodynamics. J. Fluid Mech..

[5] Esmaeeli A., Sharifi P. (2011). Transient electrohydrodynamics of a liquid drop. Phys. Rev. E.

[6] Feng J.Q., Scott T.C. (1996). A computational analysis of electrohydrodynamics of a leaky dielectric drop in an electric field. J. Fluid Mech..

[7] Torza S., Cox R., Mason S. (1971). Electrohydrodynamic deformation and bursts of liquid drops. Phil. Trans. R. Soc. Lond. A.

[8] Bentenitis N., Krause S. (2005). Droplet deformation in dc electric fields: The extended leaky dielectric model. Langmuir.

[9] Zhang J., Zahn J.D., Lin H. (2013). Transient solution for droplet deformation under electric fields. Phys. Rev. E.

[10] Zholkovskij E.K., Masliyah J.H., Czarnecki J. (2002). An electrokinetic model of drop deformation in an electric field. J. Fluid Mech..

[11] Moriya S., Adachi K., Kotaka T. (1986). Deformation of droplets suspended in viscous media in an electric field. 1. Rate of deformation. Langmuir.

[12] Zabarankin M. (2020). Small deformation theory for two leaky dielectric drops in a uniform electric field. Proc. R. Soc. A Math. Phys. Eng. Sci..

[13] Abbasi M.S., Song R., Kim H., Lee J. (2019). Multimodal breakup of a double emulsion droplet under an electric field. Soft Matter.

[14] Abbasi M.S., Song R., Kim J., Lee J. (2017). Electro-hydrodynamic behavior and interface instability of double emulsion droplets under high electric field. J. Electrost..

[15] Abbasi M.S., Song R., Kim S.M., Kim H., Lee J. (2019). Mono-emulsion droplet stretching under direct current electric field. Soft Matter.

[16] Abbasi M.S., Song R., Lee J. (2019). Breakups of an encapsulated surfactant-laden aqueous droplet under a DC electric field. Soft Matter.

[17] Bararnia H., Ganji D. (2013). Breakup and deformation of a falling droplet under high voltage electric field. Adv. Powder Technol..

[18] Brosseau Q., Vlahovska P.M. (2017). Streaming from the Equator of a Drop in an External Electric Field. Phys. Rev. Lett..

[19] Collins R.T., Jones J.J., Harris M.T., Basaran O.A. (2008). Electrohydrodynamic tip streaming and emission of charged drops from liquid cones. Nat. Phys..

[20] Das S., Thaokar R.M. (2018). Large-deformation electrohydrodynamics of an elastic capsule in a DC electric field. J. Fluid Mech..

[21] Dommersnes P., Rozynek Z., Mikkelsen A., Castberg R., Kjerstad K., Hersvik K., Fossum J.O. (2013). Active structuring of colloidal armour on liquid drops. Nat. Commun..

[22] Dubash N., Mestel A.J. (2007). Breakup behavior of a conducting drop suspended in a viscous fluid subject to an electric field. Phys. Fluids.

[23] Eggleton C.D., Tsai T.-M., Stebe K.J. (2001). Tip streaming from a drop in the presence of surfactants. Phys. Rev. Lett..

[24] Ha J.-W., Yang S.-M. (2000). Deformation and breakup of Newtonian and non-Newtonian conducting drops in an electric field. J. Fluid Mech..

[25] Ha J.-W., Yang S.-M. (1999). Breakup of a multiple emulsion drop in a uniform electric field. J. Colloid Interface Sci..

[26] Karyappa R.B., Deshmukh S.D., Thaokar R.M. (2014). Breakup of a conducting drop in a uniform electric field. J. Fluid Mech..

[27] Luo X., Yan H., Huang X., Yang D., Wang J., He L. (2017). Breakup characteristics of aqueous droplet with surfactant in oil under direct current electric field. J. Colloid Interface Sci..

[28] Mikkelsen A., Dommersnes P., Rozynek Z., Gholamipour-Shirazi A., Carvalho M.d.S., Fossum J.O.J.M. (2017). Mechanics of Pickering drops probed by electric Field–Induced Stress. Materials.

[29] Mikkelsen A., Wojciechowski J., Rajňák M., Kurimský J., Khobaib K., Kertmen A., Rozynek Z.J.M. (2017). Electric field-driven assembly of sulfonated polystyrene microspheres. Materials.

[30] Spasic A.M., Jovanovic J.M., Manojlovic V., Jovanovic M. (2016). Breaking of double emulsions based on electrohydrodynamics principles. J. Colloid Interface Sci..

[31] Ahn K., Kerbage C., Hunt T.P., Westervelt R., Link D.R., Weitz D. (2006). Dielectrophoretic manipulation of drops for high-speed microfluidic sorting devices. Appl. Phys. Lett..

[32] Fernández De La Mora J. (2007). The fluid dynamics of Taylor cones. Annu. Rev. Fluid Mech..

[33] Reznik S., Yarin A., Zussman E., Bercovici L. (2006). Evolution of a compound droplet attached to a core-shell nozzle under the action of a strong electric field. Phys. Fluid.

[34] Sankaran S., Saville D. (1993). Experiments on the stability of a liquid bridge in an axial electric field. Phys. Fluids A Fluid Dyn..

[35] Geng H., Feng J., Stabryla L.M., Cho S.K. (2017). Dielectrowetting manipulation for digital microfluidics: Creating, transporting, splitting, and merging of droplets. Lab Chip.

[36] Jia Y., Ren Y., Hou L., Liu W., Deng X., Jiang H. (2017). Sequential coalescence enabled two-step microreactions in triple-core double-emulsion droplets triggered by an electric field. Small.

[37] Liu L., Yang J.-P., Ju X.-J., Xie R., Liu Y.-M., Wang W., Zhang J.-J., Niu C.H., Chu L.-Y. (2011). Monodisperse core-shell chitosan microcapsules for pH-responsive burst release of hydrophobic drugs. Soft Matter.

[38] Loscertales I.G., Barrero A., Guerrero I., Cortijo R., Marquez M., Ganan-Calvo A. (2002). Micro/nano encapsulation via electrified coaxial liquid jets. Science.

[39] Mura S., Nicolas J., Couvreur P. (2013). Stimuli-responsive nanocarriers for drug delivery. Nat. Mater..

[40] Seifert T., Sowade E., Roscher F., Wiemer M., Gessner T., Baumann R.R. (2015). Additive manufacturing technologies compared: Morphology of deposits of silver ink using inkjet and aerosol jet printing. Ind. Eng. Chem. Res..

[41] Song R., Abbasi M.S., Lee J. (2019). Fabrication of 3D printed modular microfluidic system for generating and manipulating complex emulsion droplets. Microfluid. Nanofluid..

[42] Tucker-Schwartz A.K., Bei Z., Garrell R.L., Jones T.B. (2010). Polymerization of Electric Field-Centered Double Emulsion Droplets to Create Polyacrylate Shells. Langmuir.

[43] Xie J., Jiang J., Davoodi P., Srinivasan M.P., Wang C.-H. (2015). Electrohydrodynamic atomization: A two-decade effort to produce and process micro-/nanoparticulate materials. Chem. Eng. Sci..

[44] Bhaumik S.K., Roy R., Chakraborty S., DasGupta S. (2014). Low-voltage electrohydrodynamic micropumping of emulsions. Sens. Actuators B Chem..

[45] Wehking J.D., Kumar R. (2015). Droplet actuation in an electrified microfluidic network. Lab Chip.

[46] Xi H.-D., Guo W., Leniart M., Chong Z.Z., Tan S.H. (2016). AC electric field induced droplet deformation in a microfluidic T-junction. Lab Chip.

[47] Guan X., Hou L., Ren Y., Deng X., Lang Q., Jia Y., Hu Q., Tao Y., Liu J., Jiang H. (2016). A dual-core double emulsion platform for osmolarity-controlled microreactor triggered by coalescence of encapsulated droplets. Biomicrofluidics.

[48] Huo M., Guo Y.J.P. (2020). Electric Field Enhances Shear Resistance of Polymer Melts via Orientational Polarization in Microstructures. Polymers.

[49] Im D.J., Noh J., Moon D., Kang I.S. (2011). Electrophoresis of a charged droplet in a dielectric liquid for droplet actuation. Anal. Chem..

[50] Lecuyer S., Ristenpart W., Vincent O., Stone H. (2008). Electrohydrodynamic size stratification and flow separation of giant vesicles. Appl. Phys. Lett..

[51] Li M., Li D. (2016). Vortices around Janus droplets under externally applied electrical field. Microfluid. Nanofluid..

[52] Nguyen V.D., Byun D. (2009). Mechanism of electrohydrodynamic printing based on ac voltage without a nozzle electrode. Appl. Phys. Lett..

[53] Saville D.A. (1997). Electrohydrodynamics: The Taylor-Melcher Leaky Dielectric Model. Annu. Rev. Fluid Mech..

[54] Schnitzer O., Yariv E. (2015). The Taylor–Melcher leaky dielectric model as a macroscale electrokinetic description. J. Fluid Mech..

[55] Bazant M.Z. (2015). Electrokinetics meets electrohydrodynamics. J. Fluid Mech..

[56] Mori Y., Young Y.N. (2018). From electrodiffusion theory to the electrohydrodynamics of leaky dielectrics through the weak electrolyte limit. J. Fluid Mech..

[57] Tomar G., Gerlach D., Biswas G., Alleborn N., Sharma A., Durst F., Welch S.W.J., Delgado A. (2007). Two-phase electrohydrodynamic simulations using a volume-of-fluid approach. J. Comput. Phys..

[58] Emdadi M., Pournaderi P. (2020). Numerical simulation of conducting droplet impact on a surface under an electric field. Acta Mech..

[59] Lac E., Homsy G. (2007). Axisymmetric deformation and stability of a viscous drop in a steady electric field. J. Fluid Mech..

[60] Lanauze J.A., Walker L.M., Khair A.S. (2015). Nonlinear electrohydrodynamics of slightly deformed oblate drops. J. Fluid Mech..

[61] Sherwood J. (1988). Breakup of fluid droplets in electric and magnetic fields. J. Fluid Mech..

[62] Fernández A., Tryggvason G., Che J., Ceccio S.L. (2005). The effects of electrostatic forces on the distribution of drops in a channel flow: Two-dimensional oblate drops. Phys. Fluids.

[63] Haywood R., Renksizbulut M., Raithby G. (1991). Transient deformation of freely-suspended liquid droplets in electrostatic fields. AIChE J..

[64] Olsson E., Kreiss G. (2005). A conservative level set method for two phase flow. J. Comput. Phys..

[65] Olsson E., Kreiss G., Zahedi S. (2007). A conservative level set method for two phase flow II. J. Comput. Phys..

[66] Paknemat H., Pishevar A., Pournaderi P. (2012). Numerical simulation of drop deformations and breakup modes caused by direct current electric fields. Phys. Fluids.

[67] Soni P., Juvekar V.A., Naik V.M. (2013). Investigation on dynamics of double emulsion droplet in a uniform electric field. J. Electrost..

[68] Jiang Z., Gan Y., Luo Y. (2020). Effect of viscosity ratio on the dynamic response of droplet deformation under a steady electric field. Phys. Fluids.

[69] Huang X., He L., Luo X., Yin H., Yang D. (2019). Deformation and coalescence of water droplets in viscous fluid under a direct current electric field. Int. J. Multiphas. Flow.

[70] Huang X., He L., Luo X., Yin H. (2020). Droplet dynamic response in low-viscosity fluid subjected to a pulsed electric field and an alternating electric field. AIChE J..

[71] Ha J.-W., Yang S.-M. (1998). Effect of nonionic surfactant on the deformation and breakup of a drop in an electric field. J. Colloid Interface Sci..

[72] Ha J.-W., Yang S.-M. (1995). Effects of surfactant on the deformation and stability of a drop in a viscous fluid in an electric field. J. Colloid Interface Sci..

[73] Gu Y., Li D. (1998). Electric charge on small silicone oil droplets dispersed in ionic surfactant solutions. J. Colloids Surf. A Physicochem. Eng. Asp..

[74] Edwards M., Wu X., Wu J.-S., Huang J., H K. (1998). Electric-field effects on a droplet microemulsion. Phys. Rev. E.

[75] Mousavichoubeh M., Shariaty-Niassar M., Ghadiri M. (2011). The effect of interfacial tension on secondary drop formation in electro-coalescence of water droplets in oil. Chem. Eng. Sci..

[76] Thiam A.R., Bremond N., Bibette J. (2011). Adhesive emulsion bilayers under an electric field: From unzipping to fusion. Phys. Rev. Lett..

[77] Lee S.-H., Nguyen X.H., Ko H.S. (2012). Study on droplet formation with surface tension for electrohydrodynamic inkjet nozzle. J. Mech. Sci. Technol..

[78] Nganguia H., Young Y.-N., Vlahovska P.M., Blawzdziewicz J., Zhang J., Lin H. (2013). Equilibrium electro-deformation of a surfactant-laden viscous drop. Phys. Fluids.

[79] Wuzhang J., Song Y., Sun R., Pan X., Li D. (2015). Electrophoretic mobility of oil droplets in electrolyte and surfactant solutions. Electrophoresis.

[80] He L., Yan H., Luo X., Wang J., Huang X., Cao J., Yang D. (2016). An experimental investigation on the deformation of alkali-surfactant-polymer droplet under AC electric field. Colloid Polym. Sci..

[81] Tuček J., Beránek P., Vobecká L., Slouka Z., Přibyl M. (2016). Electric field driven addressing of oil-in-water droplets in the presence of gradients of ionic and nonionic surfactants. IEEE Trans. Ind. Appl..

[82] Mandal S., Bandopadhyay A., Chakraborty S. (2016). Dielectrophoresis of a surfactant-laden viscous drop. Phys. Fluids.

[83] Luo X., Huang X., Yan H., Yang D., Wang J., He L. (2018). Breakup modes and criterion of droplet with surfactant under direct current electric field. Chem. Eng. Res. Des. Stud..

[84] Ervik Å., Penne T.E., Hellesø S.M., Munkejord S.T., Müller B. (2018). Influence of surfactants on the electrohydrodynamic stretching of water drops in oil. Int. J. Multiphas. Flow.

[85] Poddar A., Mandal S., Bandopadhyay A., Chakrabort S. (2018). Sedimentation of a surfactant-laden drop under the influence of an electric field. arXiv.

[86] Sorgentone C., Tornberg A.-K., Vlahovska P.M. (2019). A 3D boundary integral method for the electrohydrodynamics of surfactant-covered drops. J. Comput. Phys..

[87] Poddar A., Mandal S., Bandopadhyay A., Chakraborty S. (2019). Electrorheology of a dilute emulsion of surfactant-covered drops. J. Fluid Mech..

[88] Ahmad K., Ho C., Fong W., Toji D. (1996). Properties of palm oil-in-water emulsions stabilized by nonionic emulsifiers. J. Colloid Interface Sci..

[89] Mandal S., Ghosh U., Chakraborty S. (2016). Effect of surfactant on motion and deformation of compound droplets in arbitrary unbounded Stokes flows. J. Fluid Mech..

[90] Das D., Saintillan D. (2017). Electrohydrodynamics of viscous drops in strong electric fields: Numerical simulations. J. Fluid Mech..

[91] Ouriemi M., Vlahovska P.M. (2014). Electrohydrodynamics of particle-covered drops. J. Fluid Mech..

[92] Dommersnes P., Mikkelsen A., Fossum J.O. (2016). Electro-hydrodynamic propulsion of counter-rotating Pickering drops. Eur. Phys. J. Spec. Top..

[93] Behjatian A., Esmaeeli A. (2015). Transient electrohydrodynamics of compound drops. Acta Mech..

[94] Schoeler A.M., Josephides D.N., Chaurasia A.S., Sajjadi S., Mesquida P. (2014). Electrophoretic manipulation of multiple-emulsion droplets. Appl. Phys. Lett..

[95] Soni P., Dixit D., Juvekar V.A. (2017). Effect of conducting core on the dynamics of a compound drop in an AC electric field. Phys. Fluids.

[96] O’Konski C.T., Thacher H.C. (1953). The distortion of aerosol droplets by an electric field. J. Phys. Chem..

[97] Allan R., Mason S. (1962). Particle behaviour in shear and electric fields II. Rigid rods and spherical doublets. Proc. R. Soc. Lond. Ser. A Math. Phys. Sci..

[98] Jackson J.D. (1998). Classical Electrodynamics.

[99] Stratton J.A. (2007). Electromagnetic Theory.

[100] Lamb H.J.P. (1932). Hydrodynamics.

[101] Esmaeeli A., Halim M.A. (2018). Electrohydrodynamics of a liquid drop in AC electric fields. Acta Mech..

[102] Dubash N., Mestel A. (2007). Behaviour of a conducting drop in a highly viscous fluid subject to an electric field. J. Fluid Mech..

[103] Lanauze J.A., Walker L.M., Khair A.S. (2013). The influence of inertia and charge relaxation on electrohydrodynamic drop deformation. Phys. Fluids.

[104] Nganguia H., Young Y.-N., Layton A., Lai M.-C., Hu W.-F. (2016). Electrohydrodynamics of a viscous drop with inertia. Phys. Rev. E.

[105] Vizika O., Saville D.A. (1992). The electrohydrodynamic deformation of drops suspended in liquids in steady and oscillatory electric fields. J. Fluid Mech..

[106] Jalaal M., Khorshidi B., Esmaeilzadeh E. (2010). An experimental study on the motion, deformation and electrical charging of water drops falling in oil in the presence of high voltage D.C. electric field. Exp. Therm. Fluid Sci..

[107] Mandal S., Chakrabarti S., Chakraborty S. (2017). Effect of nonuniform electric field on the electrohydrodynamic motion of a drop in Poiseuille flow. Phys. Fluids.

[108] Mandal S., Sinha S., Bandopadhyay A., Chakraborty S. (2018). Drop deformation and emulsion rheology under the combined influence of uniform electric field and linear flow. J. Fluid Mech..

[109] Esmaeeli A., Behjatian A. (2020). Transient electrohydrodynamics of a liquid drop at finite Reynolds numbers. J. Fluid Mech..

[110] Berg G., Lundgaard L.E., Abi-Chebel N. (2010). Electrically stressed water drops in oil. Chem. Eng. Process. Process Intensif..

[111] Gong H., Peng Y., Yang Z., Shang H., Zhang X. (2015). Stable deformation of droplets surface subjected to a high-voltage electric field in oil. Colloids Surf. A Physicochem. Eng. Asp..

[112] Esmaeeli A. (2018). Transient electrohydrodynamics of a liquid drop in AC electric fields. Eur. Phys. J. E.

[113] Collins R.T., Sambath K., Harris M.T., Basaran O.A. (2013). Universal scaling laws for the disintegration of electrified drops. Proc. Natl. Acad. Sci. USA.

[114] Gawande N., Mayya Y., Thaokar R. (2020). Jet and progeny formation in the Rayleigh breakup of a charged viscous drop. J. Fluid Mech..

[115] Sengupta R., Walker L.M., Khair A.S. (2017). The role of surface charge convection in the electrohydrodynamics and breakup of prolate drops. J. Fluid Mech..

[116] Ha J.-W., Yang S.-M. (2000). Electrohydrodynamics and electrorotation of a drop with fluid less conductive than that of the ambient fluid. Phys. Fluids.

[117] Salipante P.F., Vlahovska P.M. (2010). Electrohydrodynamics of drops in strong uniform dc electric fields. Phys. Fluids.

[118] Salipante P.F., Vlahovska P.M. (2013). Electrohydrodynamic rotations of a viscous droplet. Phys. Rev. E.

[119] Vlahovska P.M. (2016). Electrohydrodynamic instabilities of viscous drops. Phys. Rev. Fluids.

[120] Masliyah J.H., Bhattacharjee S. (2006). Electrokinetic and Colloid Transport Phenomena.

[121] Abbasi M.S., Farooq H., Ali H., Kazim A.H., Nazir R., Shabbir A., Cho S., Song R., Lee J. (2020). Deformation of Emulsion Droplet with Clean and Particle-Covered Interface under an Electric Field. Materials.

[122] Ouriemi M., Vlahovska P.M. (2015). Electrohydrodynamic Deformation and Rotation of a Particle-Coated Drop. Langmuir.

[123] Ha J.-W., Yang S.-M. (2000). Electrohydrodynamic effects on the deformation and orientation of a liquid capsule in a linear flow. Phys. Fluids.

[124] Nudurupati S., Janjua M., Aubry N., Singh P.J.E. (2008). Concentrating particles on drop surfaces using external electric fields. Electrophoresis.

[125] Nudurupati S., Janjua M., Singh P., Aubry N. (2010). Effect of parameters on redistribution and removal of particles from drop surfaces. Soft Matter.

[126] Hwang K., Singh P., Aubry N. (2010). Destabilization of Pickering emulsions using external electric fields. Electrophoresis.

[127] Chen G., Tan P., Chen S., Huang J., Wen W., Xu L. (2013). Coalescence of pickering emulsion droplets induced by an electric field. Phys. Rev. Lett..

[128] Cui M., Emrick T., Russell T.P. (2013). Stabilizing liquid drops in nonequilibrium shapes by the interfacial jamming of nanoparticles. Science.

[129] Rozynek Z., Dommersnes P., Mikkelsen A., Michels L., Fossum J. (2014). Electrohydrodynamic controlled assembly and fracturing of thin colloidal particle films confined at drop interfaces. Eur. Phys. J. Spec. Top..

[130] Rozynek Z., Mikkelsen A., Dommersnes P., Fossum J.O. (2014). Electroformation of Janus and patchy capsules. Nat. Commun..

[131] Rozynek Z., Castberg R., Kalicka A., Jankowski P., Garstecki P. (2015). Electric field manipulation of particles in leaky dielectric liquids. Arch. Mech..

[132] Dommersnes P., Fossum J. (2016). Surface structuring of particle laden drops using electric fields. Eur. Phys. J. Spec. Top..

[133] Mikkelsen A., Rozynek Z., Khobaib K., Dommersnes P., Fossum J.O. (2017). Transient deformation dynamics of particle laden droplets in electric field. Colloids Surf. A Physicochem. Eng. Asp..

[134] Mikkelsen A., Khobaib K., Eriksen F.K., Måløy K.J., Rozynek Z. (2018). Particle-covered drops in electric fields: Drop deformation and surface particle organization. Soft Matter.

[135] Amah E., Shah K., Fischer I., Singh P. (2016). Electrohydrodynamic manipulation of particles adsorbed on the surface of a drop. Soft Matter.

[136] Teigen K.E., Song P., Lowengrub J., Voigt A. (2011). A diffuse-interface method for two-phase flows with soluble surfactants. J. Comput. Phys..

[137] Yeh C.-H., Lee M.-H., Lin Y.-C. (2012). Using an electro-spraying microfluidic chip to produce uniform emulsions under a direct-current electric field. Microfluid. Nanofluid..

[138] Renardy Y.Y., Renardy M., Cristini V. (2002). A new volume-of-fluid formulation for surfactants and simulations of drop deformation under shear at a low viscosity ratio. Eur. J. Mech. B Fluids.

[139] Ervik Å., Hellesø S.M., Munkejord S.T., Müller B. Experimental and Computational Studies of Water Drops Falling through Model Oil with Surfactant and Subjected to an Electric Field. Proceedings of the 18th IEEE International Conference on Dielectric Liquids (ICDL).

[140] Teigen K.E., Munkejord S.T. (2009). Sharp-interface simulations of drop deformation in electric fields. IEEE Trans. Dielectr. Electr. Insul..

[141] Enayati M., Ahmad Z., Stride E., Edirisinghe M. (2010). One-step electrohydrodynamic production of drug-loaded micro- and nanoparticles. J. R. Soc. Interface.

[142] Enayati M., Chang M.-W., Bragman F., Edirisinghe M., Stride E.J.C. (2011). Electrohydrodynamic preparation of particles, capsules and bubbles for biomedical engineering applications. Colloids Surf. A Physicochem. Eng. Asp..

[143] Behjatian A., Esmaeeli A. (2013). Electrohydrodynamics of a compound drop. Phys. Rev. E.

[144] Soni P., Thaokar R.M., Juvekar V.A. (2018). Electrohydrodynamics of a concentric compound drop in an AC electric field. Phys. Fluids.

[145] Gouz H.N., Sadhal S.S. (1989). Fluid dynamics and stability analysis of a compound droplet in an electric field. Q. J. Mech. Appl. Math..

[146] Ha J.-W., Yang S.-M. (1999). Fluid dynamics of a double emulsion droplet in an electric field. Phys. Fluids.

[147] Tsukada T., Mayama J., Sato M., Hozawa M. (1997). Theoretical and Experimental Studies on the Behavior of a Compound Drop under a Uniform DC Electric Field. J. Chem. Eng. Jpn..

[148] Bei Z., Jones T.B., Harding D.R. (2010). Electric field centering of double-emulsion droplets suspended in a density gradient. Soft Matter.

[149] Bei Z.-M., Jones T., Tucker-Schwartz A., Harding D. (2008). Electric field mediated droplet centering. Appl. Phys. Lett..

[150] Bei Z.-M., Jones T., Tucker-Schwartz A. (2009). Forming concentric double-emulsion droplets using electric fields. J. Electrost..

[151] Santra S., Das S., Chakraborty S. (2020). Electrically modulated dynamics of a compound droplet in a confined microfluidic environment. J. Fluid Mech..

[152] Santra S., Das S., Chakraborty S. (2019). Electric field-induced pinch-off of a compound droplet in Poiseuille flow. Phys. Fluids.

[153] Pan Z.H., Men Y.F., Senapati S., Chang H.C. (2018). Immersed AC electrospray (iACE) for monodispersed aqueous droplet generation. Biomicrofluidics.

[154] Yin S., Huang Y., Wong T.N., Chong W.H., Ooi K.T. (2019). Electric Scissors for Precise Generation of Organic Droplets in Microfluidics: A Universal Approach that Goes beyond Surface Wettability. J. Phys. Chem. C.

[155] Azizian P., Azarmanesh M., Dejam M., Mohammadi M., Shamsi M., Sanati-Nezhad A., Mohamad A.A. (2019). Electrohydrodynamic formation of single and double emulsions for low interfacial tension multiphase systems within microfluidics. Chem. Eng. Sci..

[156] Wang H., Zhao Z., Liu Y.X., Shao C.M., Bian F.K., Zhao Y.J. (2018). Biomimetic enzyme cascade reaction system in microfluidic electrospray microcapsules. Sci. Adv..

[157] Wu D., Yu Y.R., Zhao C., Shou X., Piao Y., Zhao X., Zhao Y.J., Wang S.Q. (2019). NK-Cell-Encapsulated Porous Microspheres via Microfluidic Electrospray for Tumor Immunotherapy. ACS Appl. Mater. Interfaces.

[158] Wang D.B., Wang J.F., Yongphet P., Wang X.Y., Zuo Z.W., Li B., Zhang W. (2020). Experimental study on electric-field-induced droplet generation and breakup in an immiscible medium. Exp. Fluids.

[159] Lee K.H., Yang G.L.Z., Wyslouzil B.E., Winter J.O. (2019). Electrohydrodynamic Mixing-Mediated Nanoprecipitation for Polymer Nanoparticle Synthesis. ACS Appl. Polym. Mater..

[160] Su Y., Yu T., Wang G., Zhang C., Liu Z. (2019). Numerical simulation of electrohydrodynamics of a compound drop based on the ternary phase field method. Sci. Prog..

[161] Lee S.J., Kang J.Y., Choi W., Kwak R. (2020). Simultaneous electric production and sizing of emulsion droplets in microfluidics. Soft Matter.

[162] Rozynek Z., Khobaib K., Mikkelsen A. (2019). Opening and Closing of Particle Shells on Droplets via Electric Fields and Its Applications. ACS Appl. Mater. Interfaces.

[163] Li M.Q., Li D.Q. (2016). Redistribution of charged aluminum nanoparticles on oil droplets in water in response to applied electrical field. J. Nanoparticle Res..

[164] Brosseau Q., Hickey G., Vlahovska P.M. (2017). Electrohydrodynamic Quincke rotation of a prolate ellipsoid. Phys. Rev. Fluids.

[165] Pradillo G.E., Karani H., Vlahovska P.M. (2019). Quincke rotor dynamics in confinement: Rolling and hovering. Soft Matter.

[166] Jákli A., Senyuk B., Liao G., Lavrentovich O.D. (2008). Colloidal micromotor in smectic A liquid crystal driven by DC electric field. Soft Matter.

[167] Das D., Lauga E. (2019). Active Particles Powered by Quincke Rotation in a Bulk Fluid. Phys. Rev. Lett..

[168] Zrinyi M., Nakano M. (2017). Toward Colloidal Motors. Period. Polytech.-Chem. Eng..

[169] Belovs M., Cebers A. (2020). Quincke rotation driven flows. Phys. Rev. Fluids.

[170] Nudurupati S., Janjua M., Singh P., Aubry N. (2009). Electrohydrodynamic removal of particles from drop surfaces. Phys. Rev. E.

[171] Rozynek Z., Jozefczak A. (2016). Patchy colloidosomes—An emerging class of structures. Eur. Phys. J. Spec. Top..

[172] Jia Y.K., Ren Y., Hou L.K., Liu W.Y., Jiang T.Y., Deng X.K., Tao Y., Jiang H.Y. (2018). Electrically controlled rapid release of actives encapsulated in double-emulsion droplets. Lab Chip.

[173] Deng X.K., Ren Y.K., Hou L.K., Liu W.Y., Jiang T.Y., Jiang H.Y. (2019). Compound-Droplet-Pairs-Filled Hydrogel Microfiber for Electric-Field-Induced Selective Release. Small.

